# Population structure and genetic diversity characterization of soybean for seed longevity

**DOI:** 10.1371/journal.pone.0278631

**Published:** 2022-12-06

**Authors:** Naflath T. V., Rajendra Prasad S., Ravikumar R. L.

**Affiliations:** 1 Department of Seed Science and Technology, College of Agriculture, UAS, GKVK, Bangalore, Karnataka, India; 2 Department of Plant Biotechnology, College of Agriculture, UAS, GKVK, Bangalore, Karnataka, India; ICAR-Indian Institute of Pulses Research, INDIA

## Abstract

Seed longevity is an important trait in the context of germplasm conservation and economics of seed production. The identification of populations with high level of genetic variability for seed longevity and associated traits will become a valuable resource for superior alleles for seed longevity. In this study, Genotyping-by-sequencing (GBS)-single nucleotide polymorphism (SNP) approach, simple sequence repeats (SSR) markers and agro-morphological traits have been explored to investigate the diversity and population structure of assembled 96 genotypes. The GBS technique performed on 96 genotypes of soybean (*Glycine* max (L.) Merrill) resulted in 37,897 SNPs on sequences aligned to the reference genome sequence. The average genome coverage was 6.81X with a mapping rate of 99.56% covering the entire genome. Totally, 29,955 high quality SNPs were identified after stringent filtering and most of them were detected in non-coding regions. The 96 genotypes were phenotyped for eight quantitative and ten qualitative traits by growing in field by following augmented design. The STRUCTURE (Bayesian-model based algorithm), UPGMA (Un-weighed Pair Group Method with Arithmetic mean) and principal component analysis (PCA) approaches using SSR, SNP as well as quantitative and qualitative traits revealed population structure and diversity in assembled population. The Bayesian-model based STRUCTURE using SNP markers could effectively identify clusters with higher seed longevity associated with seed coat colour and size which were subsequently validated by UPGMA and PCA based on SSR and agro-morphological traits. The results of STRUCTURE, PCA and UPGMA cluster analysis showed high degree of similarity and provided complementary data that helped to identify genotypes with higher longevity. Six black colour genotypes, *viz*., Local black soybean, Kalitur, ACC Nos. 39, 109, 101 and 37 showed higher seed longevity during accelerated ageing. Higher coefficient of variability observed for plant height, number of pods per plant, seed yield per plant, 100 seed weight and seed longevity confirms the diversity in assembled population and its suitability for quantitative trait loci (QTL) mapping.

## Introduction

Seed longevity has an exceptional importance in the conservation of genetic resources since majority of 7.4 million gene bank accessions of plants species are stored as seeds [1]. It maintains the species without losing its genetic integrity, that can happen during regeneration of plants and holds up the global agriculture. The longevity of a seed in storage is highly varied between the species, varieties and even in different seed lots of a variety [2–5]. Soybean (*Glycine max* (L.) Merrill) is an important crop providing sustainable source of vegetable oil and high-quality protein to world population and live-stock [6]. It is not surprising that in 2021, soybeans are the largest global source of protein for live-stock and the global production of soybean has more than doubled since 2000 [7]. More than ninety countries grow soybean [8] and modern soybean varieties have been developed for high yield and wide adaptability; but low seed longevity [9, 10]. Large genetic variation in seed longevity has been reported in soybean [11–13] and observed that black seed coat colour [12, 14–16] and small seed size [17] are associated with seed longevity. It has been documented that thin and delicate seed coat and high seed oil content makes the soybean more susceptible to deterioration under dry storage [18–21]. Soybean with black seed coat colour have been used as a traditional ingredient in medical treatments in many countries with many health benefits [6, 22, 23]. However, black soybeans, mostly landraces with higher longevity have been declined in popularly and replaced by improved varieties. Therefore, identification of populations with high level of genetic variability for seed longevity and associated traits will become a valuable resource for superior alleles for seed longevity. To understand the importance of the genetic diversity for seed longevity in soybean, the genotypes with different seed coat colour, seed size, germplasm and advanced breeding lines with abundant morphological diversity were assembled. Understanding population structure and genetic diversity in this population is important which paves the way for genome wide association studies and for functional gene investigation.

Analysing the genetic diversity and population genetic structure is significant for broadening the base population and allelic diversity in crop improvement. Numerous techniques can be used to determine the genetic variations. Among them, morphological and quantitative phenotypic traits have been extensively used to determine the genetic diversity in crop plants [24–26] including soybean [27, 28]. These traits are affected by many external factors and limited in number [29–31]. Morphological markers are less effective in understanding the genetic diversity and population structure [32, 33]. Hence, molecular markers became a powerful tool for the genetic studies of soybean populations. These includes random amplified polymorphic DNA (RAPD) [34–36], simple sequence repeats (SSR) [37, 38], amplified fragment length polymorphism (AFLP) [39, 40], inter simple sequence repeats (ISSR) [35, 41] and single nucleotide polymorphism (SNP) [42–44]. Among these, microsatellite (SSR) and SNP markers are the commonly used markers in estimating the genetic diversity and population structure of different species either singly [45–49] or in combination [50–53]. These two marker systems are distributed throughout the genome while the frequency and basis of polymorphism varies [54]. The abundance of SNPs over SSRs in a genome with low sequencing cost and easy genotyping techniques facilitated the SNP marker system as an overwhelmingly useful method. Genotyping-by-sequencing (GBS) is a next generation sequencing (NGS) technique, which sequence a representative genomic region and genotype the samples based on the identified SNPs at once [45, 55]. It has been a technique of high acceptance in genomic studies [56–60] and widely used in population structure as well as genetic diversity studies [45, 61, 62].

Therefore, this research was conducted to perform a genetic diversity and population structure analysis from morphological, SSR and SNP markers on a soybean population of 96 genotypes differing in seed coat colour, seed size, growth habit, seed longevity and agro-morphological traits. The black seed coat colour and small seed size land races have been a special attraction for seed longevity. Our findings will facilitate future genome wide association mapping and functional aspects of seed longevity.

## Material and methods

### Plant material

A set of 96 soybean genotypes including 11 black, three green and 82 yellow seed coat colours (landraces, germplasm and advanced breeding lines) were included in this study (S1 Table). The genotypes were obtained from all India co-ordinated research project (AICRP) on soybean, University of agricultural sciences (UAS) Bangalore, Indian council of agricultural research, Ministry of agriculture, India, the seed material available with the first author and also at AICRP on soybean, UAS Dharwad. The field activities were conducted properly within the Indian laws and regulations at Department of plant biotechnology, UAS Bangalore. We confirm that the field studies conducted in the current study did not involve endangering indigenous or protected species.

### Phenotyping under field conditions

The genotypes were phenotyped for eight quantitative and ten qualitative traits by growing the genotypes in the field during summer 2021 at Department of Plant Biotechnology, UAS Bangalore, Karnataka, India. The 96 genotypes were grown side by side in a single row of 2.0 meter without replication using augmented block design and randomly assigned to three blocks. Each block contained 32 genotypes and 4 checks (DSB 32, JS 95–60, Hardee and DSB 33) repeated twice. All the agronomic practices were followed to raise a good crop. Five plants were randomly selected from each genotype and the measurements on the following quantitative traits were recorded. The days to flowering (DFF) was recorded as the numbers of days from sowing until 50 percent of the plants bloomed. The plant height (PH) in centimetre was measured as the distance from the base of the plant (soil surface) to tip of the primary branch at maturity. Number of pods per plant (NPP) were counted by taking pods with at least one developed seed at maturity. Five pods per plant were randomly selected to count the number of seeds per pod (NSP) and pod length (PL) in centimetre was measured form base to the tip of the matured pod. Seed yield per plant (SY) in gram was taken after sun drying the seeds to uniform moisture content (9%) and 100 seed weight (100SW in gram) was recorded by weighing 100 filled seeds. The qualitative traits *viz*., hypocotyl anthocyanin pigmentation (HP), growth habit (GH), growth type (GT), flower colour (FC), leaf shape (LS), leaf greenness (LG), stem pubescence (SP), pod pubescence (PP), pod colour (PC) and seed coat colour (SCC) were recorded by visual observation as per distinctness, uniformity and stability (DUS) guidelines on soybean [63].

The seed longevity (G%) was measured using germination percentage after accelerated ageing. Freshly harvested seeds after sun drying to nine percent moisture content (measured using digital moisture meter (Kett-Seed & Grain Moisture Tester PM-600)) was subjected to accelerated ageing treatment [64] at National seed project, UAS, Bangalore, Karnataka, India. Forty two gram seeds from all the genotypes were placed on a wire mesh screen in an ageing box filled with 40 ml distilled water and subjected to 41±0.3°C temperature and more than 95 percentage relative humidity in an ageing chamber for 72 hours. DSB 32, JS 95–60, Hardee and DSB 33 were used as check varieties. Another set of all the genotypes were placed in separate ageing boxes and kept at ambient conditions without accelerated ageing treatment as control. After ageing treatment, one hundred seeds in four replicates from treated and control boxes were subjected to germination process using between paper method as per international seed testing association (ISTA) guidelines [65] at a temperature of 30°C with a relative humidity of 90±2 percent for 5 days. The germination count was recorded and expressed in percentage.

### SNP genotyping

Two sets of genomic DNA from each genotype was isolated from fresh leaves of 10 days old seedling using CTAB method [66]. The Genotyping by sequencing [55] was outsourced to the company, Nucleome Informatics Pvt. Ltd., Hyderabad, India (https://www.nucleomeinfo.com). Genomic DNA of 0.3–0.6 μg from all the genotypes was digested using *NlaIII_HaeIII* and *MseI* restriction enzymes based on *in silico* evaluation results, and the obtained fragments were ligated with two barcoded adapters as well as P5 and P7 universal sequence followed by polymerase chain reaction (PCR) enrichment. The library was constructed by pooling the DNA fragments with desired sizes after gel electrophoresis. High-throughput pair-end-sequencing of the library was performed with a read length of 144 base pair (bp) at each end using Illumina^®^ HiSeq^TM^ 2500 platform. The base calling of the sequence was performed using CASAVA v1.8 software. The Illumina sequencing quality (Q_phred_) was calculated by using the equation, *Q_phred_* = −10 log_10_(*e*) [67], were *e* is the sequencing error rate in CASAVA version 1.8 software. The sequencing error rate or base quality is examined over the length of all sequences to detect the sites with error rate which increases with extension of sequencing reads due to the consumption of chemical reagents, laser irradiation damage of DNA, error during the sequencing cycles and high error rate of several bases at first due to the reading errors. The GC content distribution analysis was done to detect the AT or GC separation of the pair-end sequencing data. The raw data obtained from GBS was subjected to filtration by removing the reads with adapter contamination, more than 10 percent uncertain nucleotides and more than 50 percent low quality nucleotides.

After the quality check and data filtering, the GBS data was subjected to sequence assembly with reference genome (https://www.ncbi.nlm.nih.gov/assembly/GCF_000004515.6/) using Burrow-Wheeler Aligner (BWA) [68]. The SNP calling was performed using TASSEL (v. 5) GBS v2 pipeline [69]. Summary of the filtered data was computed using VCFtools [70]. The nucleotide variant annotation and effect prediction of detected SNPs was performed using SnpEff software v5.1 [71]. SnpEff annotates variants based on their genomic locations and predicts coding effects. Annotated genomic locations include intronic, untranslated region, upstream, downstream, splice site, or intergenic regions. The SNP filtering was performed in TASSEL 5 using the FilterSiteBuilderPlugin and the filtering criteria was set to 0.05 site minor allele frequency, 0.20 maximum heterozygous proportion and less than 10.00 percent missing data.

Another set of DNA, isolated from 96 genotypes was used for SSR genotyping using fifteen microsatellite markers and the primer details and standardized annealing temperature for the primers are given in S2 Table. The volume of reaction mixture was 10 μl and 38 reaction cycles were run. The PCR products were visualized in 3.5 percent agarose gel using ethidium bromide in gel documentation system. Marker scoring was done manually as 0/1 binary matrix. The presence of an allele was denoted as ‘*1*’ and absence as ‘*0*’ and the size of the amplified fragments were recorded in comparison with 1000 bp DNA ladder.

### Statistical data analysis

#### Morphological diversity, principal component analysis and clustering

The analysis of variance (ANOVA) was done using augmented-randomized complete block design for the quantitative observation of the genotypes. The angular transformed values of germination percentage were analysed using completely randomized design (CRD) in SPSS software [72]. Mean, range and percentage coefficient of variation (CV) of the traits were computed in microsoft excel. The violin plot of the quantitative traits are performed in PAST04 software [73]. The graphical representation of frequency distribution of genotypes in all the qualitative observations were computed in microsoft excel. The principal component analysis (PCA) and clustering of eight quantitative (DFF, PH, NPP, NSP, PL, SY, 100SW and G%) and ten qualitative traits (HP, GH, GT, FC, LS, LG, SP, PP, PC and SCC) was carried out in PAST04 software. A scatter plot was developed to visually assess the dissimilarity between the genotypes based on their positions in the plot depending on seed longevity and other plant morphological traits of the genotypes. The UPGMA (Un-weighed Pair Group Method with Arithmetic mean) based clustering of the genotypes was done using Euclidean distance-similarity index by taking all the phenotypic diversity including seed longevity.

#### Population structure and clustering using SNP

The population structure was analyzed by means of K values which is an assumed number of sub-populations from 1 to 10 in the population using STRUCTURE v.2.3.4 software [74]. Ten independent analyses were performed for each K value and the parameters was set as 50,000 lengths of burn-in period followed by 50,000 markov chain monte carlo (MCMC) replications after burn-in. The optimum K value was determined in Structure Harvester v 0.6.94 [75] that creates a plot of mean likelihood values per K. The actual number of sub-populations was selected based on the maximum value of delta K [74, 76].

The distance-based population structure analysis was performed using SNP markers in TASSEL (v5) software. Hierarchical clustering was carried out using UPGMA by taking pair wise dissimilarity matrix between the genotypes [77]. Nei coefficient was used to estimate the genetic distance between the genotypes [78] with 1000 bootstraps.

#### Genotype clustering using SSR markers

The UPGMA based population structure analysis was performed using fifteen SSR markers in DARwin software [79] by taking the pairwise dissimilarity index of SSR markers between the genotypes.

## Results

### SNP genotyping, population structure and clustering

**Fig 1:**
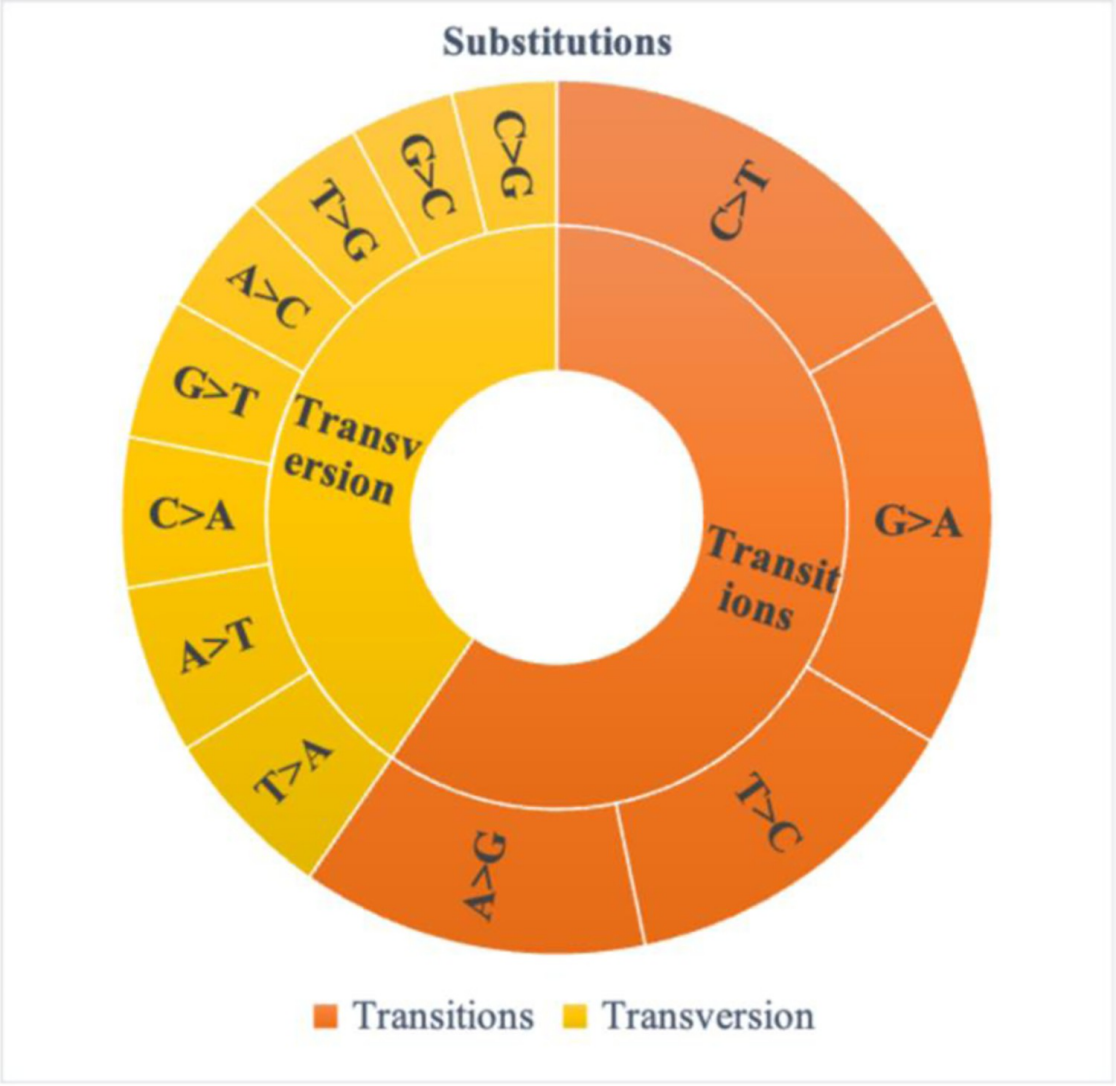
Number of transitions and transversion of nucleotide in genotyping by sequencing analysis.

**Fig 2:**
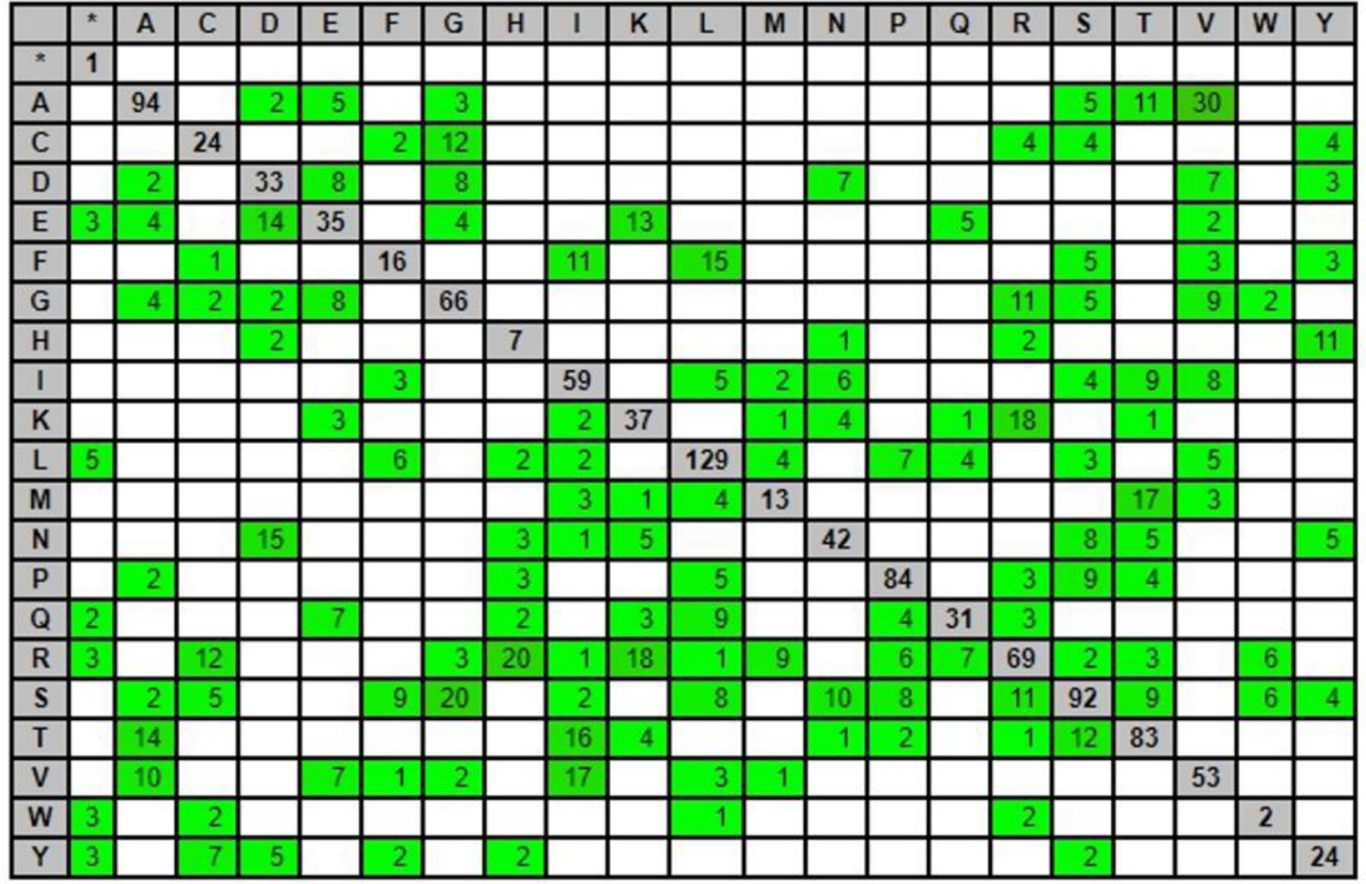
Amino acid changes of the variants in SnpEff analysis of the GBS derived SNPs. (Rows are reference amino acids and columns are changed amino acids. For example: 2 Alanine have been replaced by Aspartic acid. The increase in intensity of green colour indicate the increase in number of amino acid changes and the diagonals are indicated in grey background colour). The amino acid codes- A: Alanine; C: Cysteine; D: Aspartic acid; E: Glutamic acid; F: Phenylalanine; G: Glycine; H: Histidine; I: Isoleucine; K: Lysine; L: Leucine; M: Methionine; N: Asparagine; P: Proline; Q: Glutamine; R: Arginine; S: Serine; T: Threonine; V: Valine; W: Tryptophan; Y: Tyrosine.

Genotyping by sequencing analysis was performed on 96 diverse soybean genotypes by Illumina^®^ HiSeq^TM^ 2500 platform. Totally 28.088Gb raw data of 96 samples were sequenced from this study, with 28.0Gb clean data generated after filtering low-quality data. The raw data production for each sample ranged from 154.12 to 381.48 million reads, indicating the sufficient amount of data production. The sequencing quality for Q20 and Q30 reached 94.44% and 85.00%, respectively. The GC content ranged from 35.25% to 38.13%, fulfilling the quality standard for SNP analysis (S3 Table). For 989,192,843 bp reference genome, the mapping rate of each sample ranged from 97.77% to 99.69% with an average of 99.56%. The average depth (X) on the reference genome is in 4.60X to 8.76X range with a mean of 6.81X, while more than 1X coverage exceeds 2.87% (S4 Table). Totally 37,897 SNPs were identified during SNP calling using TASSEL (v. 5) GBS v2 pipeline. The variant rate was one variant in every 25,299 bases. The type and number of substitutions observed in the GBS analysis shows higher number of transitions (22,584) than transversion (15,273) with a ratio of 1.47 (Fig 1). Totally 39 insertions and deletions were identified from the GBS analysis. The effect by impact and functional class studied for the identified SNPs using SnpEff shows that, 97.25% SNPs were with modifier type impact in which mutations occurs in introns, intergenic, intragenic and other non-coding regions (Table 1). Only 1.67% and 1.02% variants were detected with low (variants which do not change encoded protein) and moderate (variants cause changes in protein function without affecting the protein structure) impact. Totally, 38 (0.05%) mutations were detected that cause significant changes (high impact) like frame shift, deletion of large part of chromosome or the entire exon and formation of stop codon. SnpEff detected higher percentage of point mutations or SNPs, with missense variant (59.60%) which results in change in amino acids. There are 38.94% silent mutations detected from the identified SNPs that do not cause significant change in the protein. The SNPs with nonsense effects was only 1.46% which results in formation of stop codons and subsequently produce truncate, incomplete or non-functional proteins. Various kinds of SNPs were detected from the GBS analysis of soybean genotypes including gain and loss of stop codon and start codon, respectively (Table 1). The SNPs were also detected at splice regions (0.27%), intergenic (41.96%) and intragenic (1.01%) regions. The possible amino acid changes estimated using SnpEff indicate that the detected SNPs has resulted in numerous rearrangements of the amino acid sequences of protein (Fig 2). Highest number of changes was detected in the replacement of Alanine with Valine (30) followed by Arginine with Histidine (20) and Serine with Glycine (20).

**Table 1:** SnpEff summary of variant effects by impact and functional class of GBS derived SNPs.

	Type	Count	Percent
**Effect by impact**	High	38	0.05
	Low	1,268	1.67
	Moderate	775	1.02
	Modifier	73,507	97.25
**Functional class**	Missense variant	776	59.60
	Nonsense variant	19	1.46
	Silent variant	507	38.94
**Variant type**	3_prime_UTR_variant	611	0.81
	5_prime_UTR_variant	773	1.02
	5_prime_UTR_premature_strat_codon) gain_variant	104	0.14
	Downstream_gene_variant	14,997	19.79
	Initiator_codon_variant	1	0.001
	Intergenic_region	31,809	41.96
	Intragenic_variant	766	1.01
	Intron_variant	8,579	11.32
	Non_canonical_start_codon	1	0.001
	Non_coding_transcript_exon_variant	756	0.99
	Splice_acceptor_variant	9	0.01
	Splice_donor_variant	9	0.01
	Splice_region_variant	204	0.27
	Start_lost	1	0.001
	Stop_gained	19	0.03
	Stop_retained_variant	1	0.001
	Synonymous_variant	992	1.31
	Upstream_gene_variant	15,394	20.31

The detected SNPs were then subjected to filtering by removing SNPs with less than 5 percent minor allele frequency, greater than 20 percent heterozygous proportion and greater than 10 percent missing data (Table 2). A total of 29,955 high quality SNP markers were obtained after filtering and further used for population structure analysis. The proportion of heterozygosity and average minor allele frequency of the filtered SNPs were 0.02 and 0.25, respectively. Among 29,955 SNPs identified, 26,769 SNPs were aligned to 20 chromosomes and 3,186 SNPs were found in contigs. Higher number of SNPs were aligned to chromosome-18 (10.33%) followed by chromosome-15 (8.74%) and chromosome-14 (7.46%) and least was in chromosome-10 (2.81%) (S5 Table).

**Table 2:** SNP calling and filtering summary.

	Raw SNPs	Filtered SNPs
Number of SNPs	37897	29955
Number of missing	426022	21267
Proportion of missing	0.116	0.007
Number of heterozygous	599504	60189
Proportion of heterozygous	0.16	0.02
Average minor allele frequency	0.22	0.25

**Fig 3:**
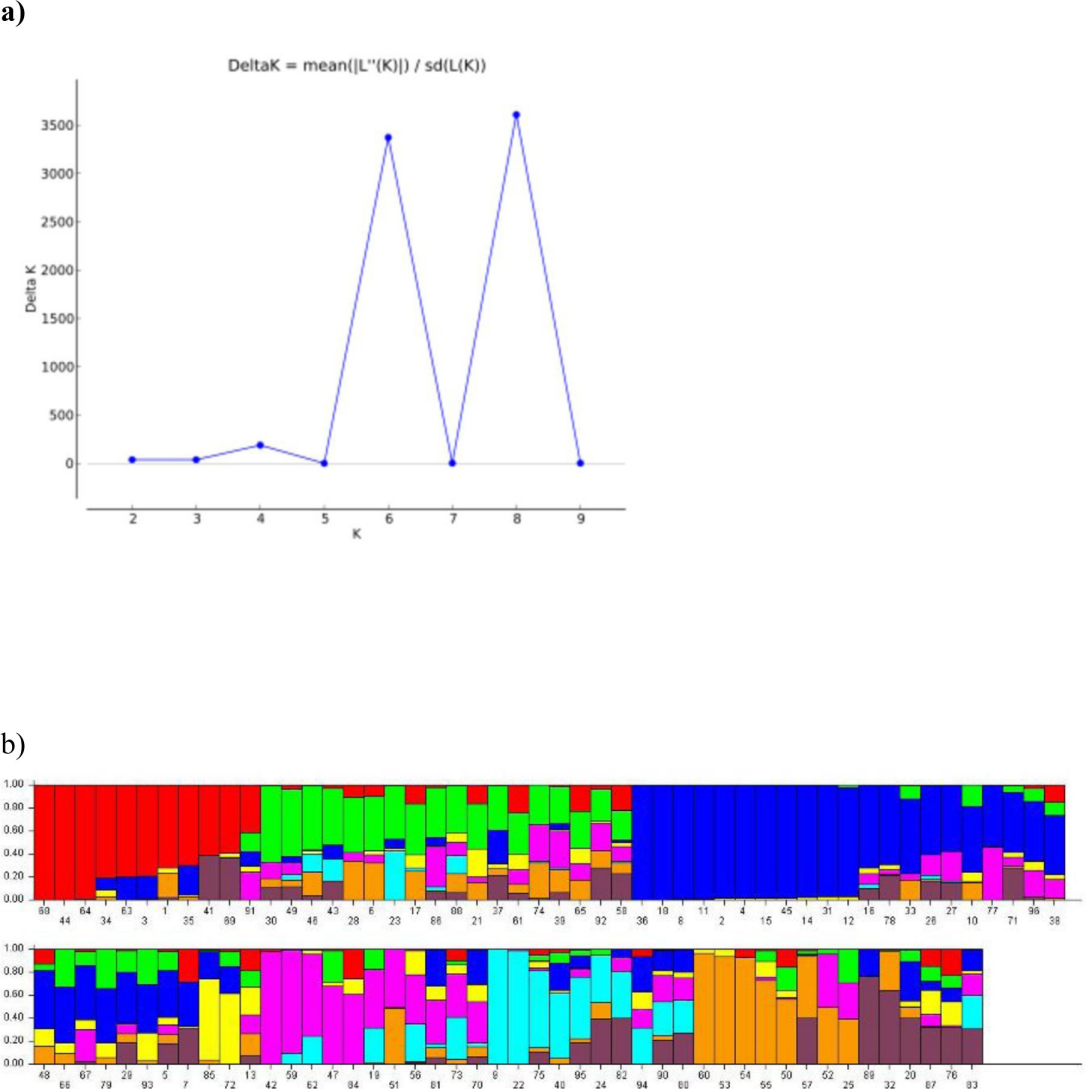
SNP marker based population structure. a) Delta K plot at different K-value based on the maximum likelihood method b) Barplot of K = 8 with genotypes in each sub-population depending on their inferred ancestry.

The structure analysis of 96 soybean genotypes was performed using 29,955 SNPs assuming 10 populations. The optimum number of clusters was estimated based on ΔK method and plateau criterion in the STRUCTURE. The maximum likelihood based ad-hoc method gave highest value of ΔK at K = 8 explaining that 96 genotypes can be grouped in to eight distinct populations (Fig 3A). The eight populations derived from structure analysis consisted of 11, 18, 29, 3, 11, 10, 8 and 6 genotypes, respectively (Fig 3B). Based on the membership fractions, *i*.*e*., inferred ancestry, the genotypes were placed in different sub-populations. The sub-population C7 consisted of seven black seeded genotypes along with one yellow which had 31% and 28% of membership to C5 and C2, respectively. Another black genotype, ACC No. 369 had 49% membership to C7 though it was placed in C5 (50%). All the genotypes in C2, C4 and C8 sub-populations had admixture from rest of the sub-populations. In addition, ΔK peak was also observed at K = 6, which indicate an additional informative population structure (S1 Fig; S6 Table).

Remarkable genetic divergence among the sub-populations derived from the population structure analysis was evident from the average genetic distance (expected heterozygosity, *He*). Highest value of *He* was detected in C4 and least was noted in C3 (Table 3). The fixation index (F_st_) resulted from structure analysis indicate genetic variance of each sub-population to the total population variance. Highest F_st_ was found in C8 (0.81), followed by C3 (0.80) and least was in C4 (0.01). The sub-population-wise average seed longevity (G%) was highest for C7 (53.74%) followed by C4 (51.30%) with 100SW of 10.86 and 10.10 g, respectively. The PH was highest in C7 (73.08) followed by C4 (57.63) and least PH belonged to C2 (39.15). Seven out of eight genotypes in C7 were black seed coat colour genotypes and had smaller seed size with higher plant height.

**Table 3:** Fixation index, expected heterozygosity and number of genotypes in eight sub groups of soybean genotypes derived from SNP population structure analysis.

Subpopulations	Fixation index (Fst)	Inferred clusters	Expected Heterozygosity (He)	No. of genotypes	Seed longevity	100 Seed weight (g)	Plant height (cm)
**C1**	0.66	0.12	0.17	11	38.22	14.16	53.07
**C2**	0.59	0.12	0.22	18	38.98	14.62	47.26
**C3**	0.80	0.26	0.08	29	39.29	13.92	39.15
**C4**	0.01	0.06	0.45	3	51.30	10.10	57.63
**C5**	0.66	0.14	0.17	11	44.48	13.87	42.00
**C6**	0.78	0.09	0.12	10	38.19	14.10	51.41
**C7**	0.79	0.12	0.11	8	53.74	10.86	73.08
**C8**	0.81	0.09	0.12	6	34.48	13.44	47.68

NOTE: C1: sub-population-1; C2: sub-population-2; C3: sub-population-3; C4: sub-population-4; C5: sub-population-5; C6: sub-population-6; C7: sub-population-7; C8: sub-population-8

**Fig 4:**
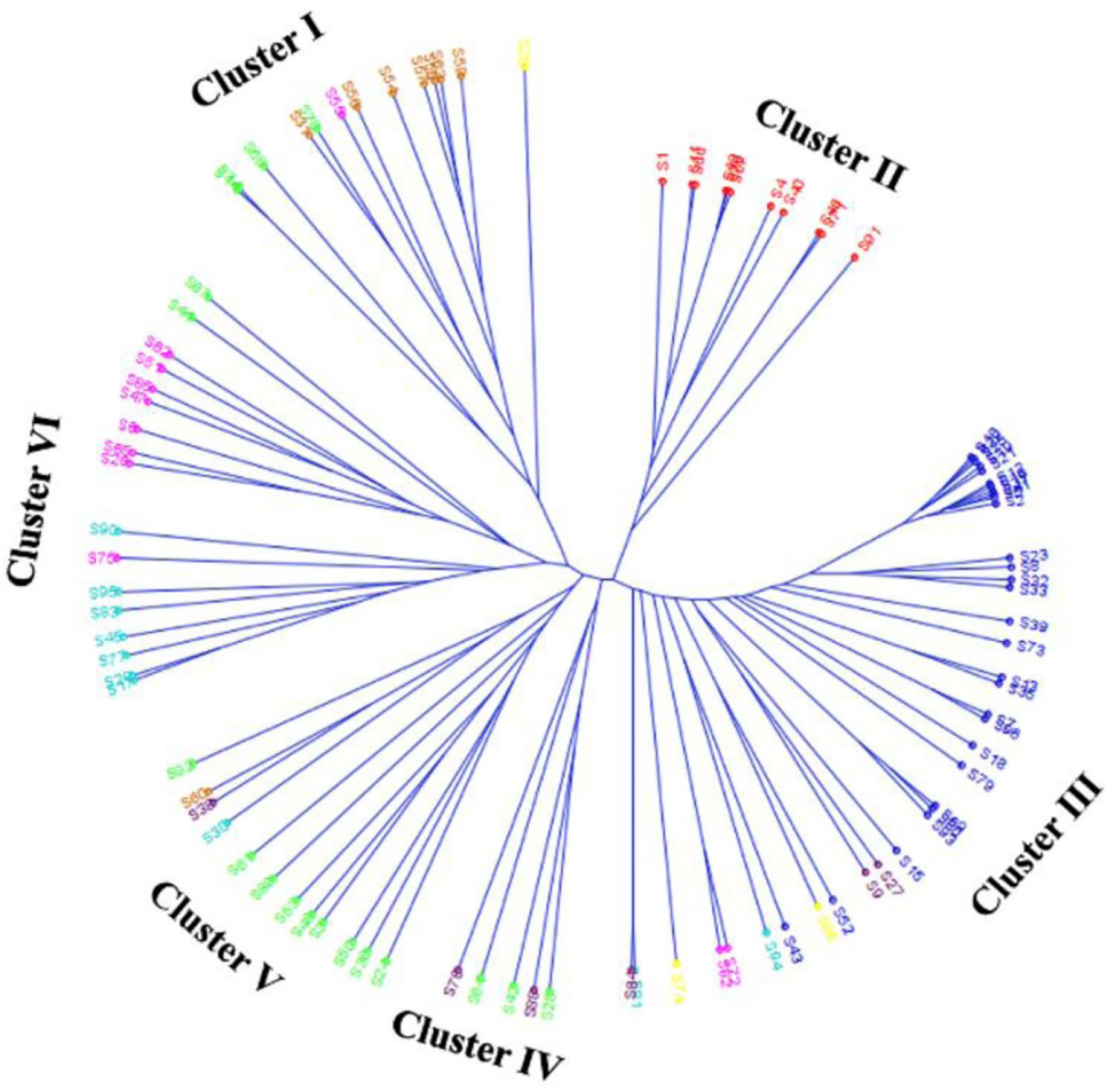
UPGMA based hierarchical clustering of genotypes using SNP markers.

In this study, UPGMA hierarchical clustering using 29,955 SNP loci was also carried out to analyse the population structure. The UPGMA divided the 96 genotypes to six distinct clusters consisting of 13, 11, 38, 5, 12 and 17 genotypes, respectively (Fig 4). The results are complementary to the STRUCTURE analysis. Out of 13 genotypes in cluster-I, seven were from C7 sub-population of STRUCTURE. Five out of seven black seed coat colour genotypes in this cluster formed a sub-cluster and BNS-5 (S20) (green) formed a solitary cluster. Interestingly, all the 11 genotypes in cluster-II were from C1 sub-population of STRUCTURE. Cluster-III consists of all the genotypes in C3 sub-population along with genotypes from other sub-populations and contain 36 yellow and 2 black (Pune 14 (S13) and Pune 30 (S35)) seed coat colour genotypes. Cluster-IV consists of yellow seed coat colour genotypes from C2 and C8. Nine out of 12 genotypes in cluster-V were the same as C2.

#### Molecular clustering using SSR marker

**Fig 5:**
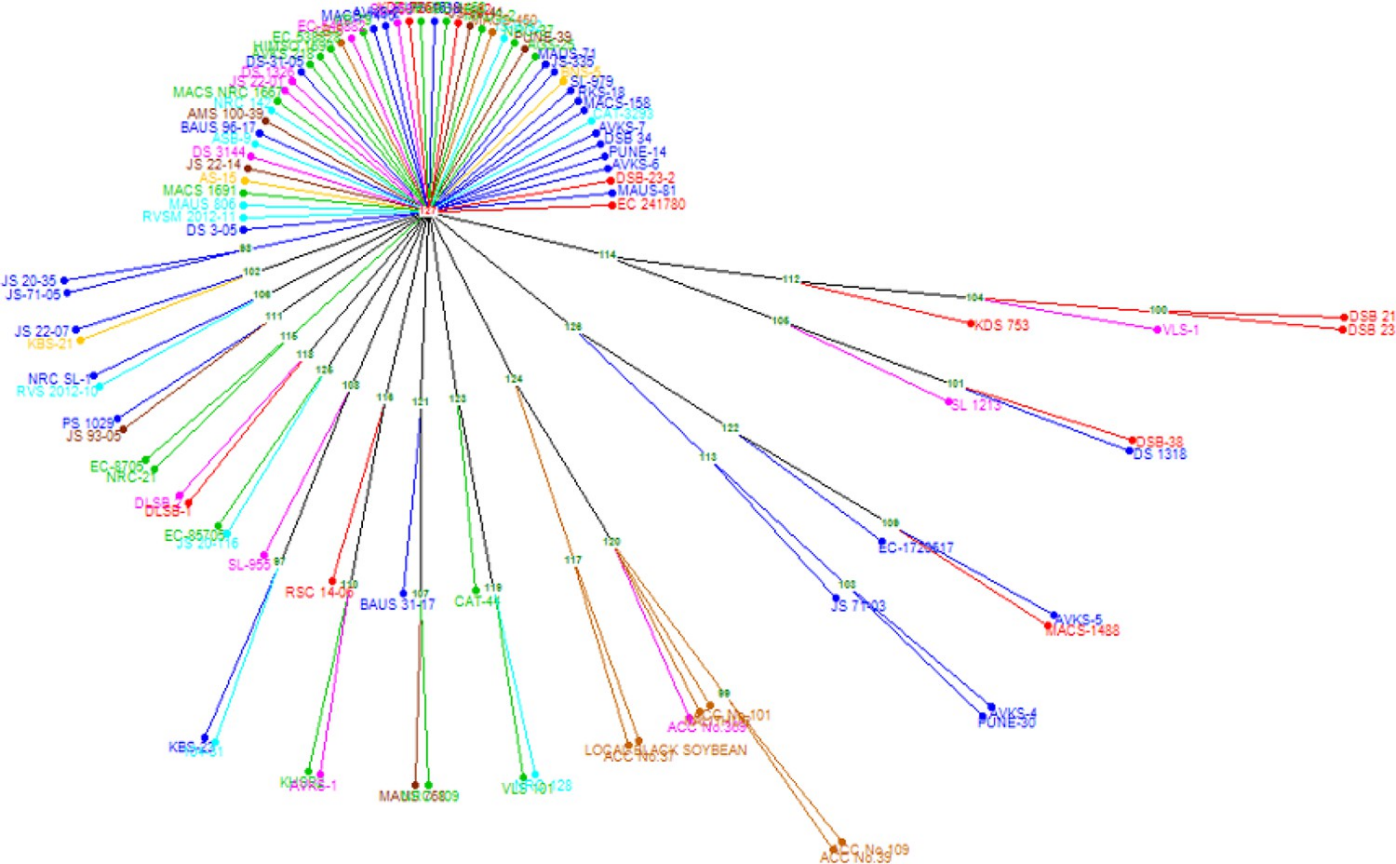
UPGMA cluster of 96 soybean genotypes using SSR markers.

The UPGMA paired hierarchical clustering of the genotypes were also performed using 15 SSR markers (Fig 5). Majority of the genotypes (50 genotypes) formed solitary clusters with only one genotype per cluster. The remaining 46 genotypes formed 14 clusters. However, the seven black seed coat colour genotypes formed one cluster in SSR genotyping along with 6 genotypes from C7 sub-population of STRUCTURE. The clustering based on SSR genotyping did not show any complementation with clustering based on SNP genotyping.

#### Phenotypic variation, principal component analysis and clustering

**Fig 6:**
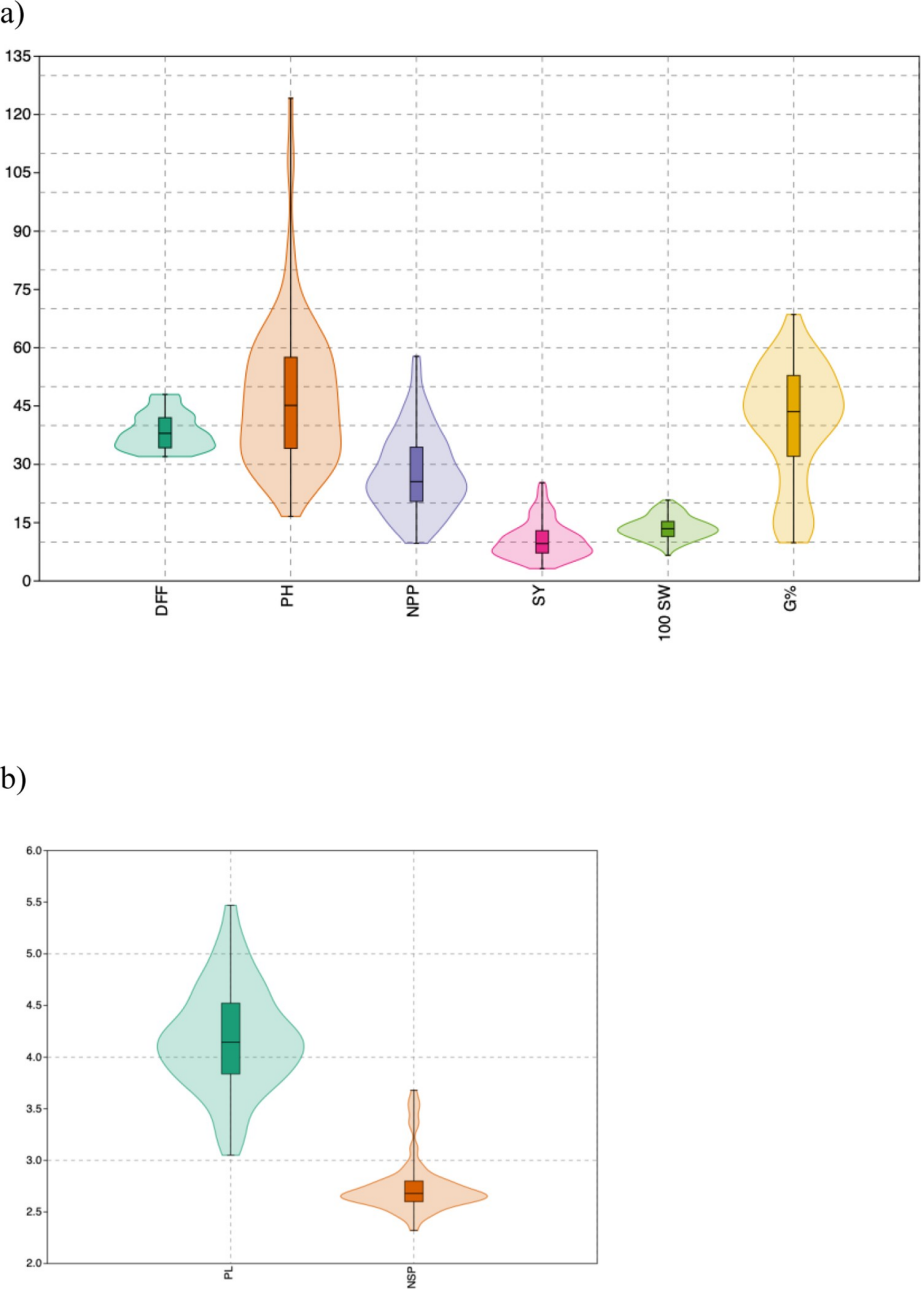
Violin plot of quantitative traits. a) Plot of Days to flowering (DFF), Plant height (PH), Number of pods per plant (NPP), Seed yield (SY), 100 Seed weight (100SW) and Seed longevity (G%) b) Plot of Pod length (PL) and Number of seeds per pod (NSP).

The genotypes were evaluated for eight quantitative traits and the analysis of variance suggested significant variation for all the traits including seed longevity (Table 4). The phenotypic variation in all the eight traits (DFF, PH, NPP, NSP, PL, SY, 100SW and G%) among genotypes is depicted by range, mean, standard error and coefficient of variation (Table 5). The violin plot of genotypes for different traits indicated normal distribution for the traits with high density of the kernel density plot towards median, except for PH and DFF (Fig 6A and 6B). In DFF, a high-density peak of the genotypes was observed at lower quartile and a slight peak at upper quartile with a bimodal distribution. The kernel density of genotypes for PH were distributed throughout the inter quartile range without a remarkable peak. The G% also showed normal distribution.

**Table 4:** Mean sum of square of genotypes and error for eight quantitative traits.

Source	Days to flowering	Plant height	Number of pods per plant	Number of seeds per pod	Pod length	Seed yield	100 Seed weight	Seed longevity
Entries (Genotypes+ Checks)	99.00	24.24	355.00	108.30	0.068	0.245	8.62	-
Blocks	2.00	0.58	0.36	11.00	0.002	0.0034	0.01	-
Checks	3.00	140.75***	288.00***	186.70***	0.003	0.039**	7.55***	-
Genotypes	20.78***	357.00***	106.70***	0.07***	0.253***	22.95***	8.59***	2037.37***
Checks vs Genotypes	1.00	4.03**	361.00***	30.50**	0.001	0.07**	14.97***	-
Error	0.25	4.00	2.00	0.002	0.002	0.04	0.01	30.84

Significance codes

***-p = 0.001

**- p = 0.01

*- p = 0.05

**Table 5:** Mean, range and coefficient of variation (CV %) of genotypes for eight quantitative traits.

Traits	Range	Mean±SE	Coefficient of Variation (%)
Days to flowering (DFF)	32.00–48.00	38.53±0.50	11.83
Plant height (cm) (PH)	16.60–124.20	47.80±1.92	39.52
Number of pods per plant (NPP)	9.67–57.80	27.92±1.05	36.98
Number of seeds per pod (NSP)	2.32–3.68	2.74±0.03	9.67
Pod length (cm) (PL)	3.05–5.47	4.17±0.05	12.03
Seed yield per plant (g) (SY)	3.16–25.28	10.52±0.20	45.52
100 seed weight (g) (100SW)	6.60–20.80	13.68±0.29	21.41
Seed longevity (G%)	3.00–86.50 (9.83–68.54)*	44.63±3.86 (40.86) *	37.72

*Values in the parenthesis are the angular transformed value

**Fig 7:**
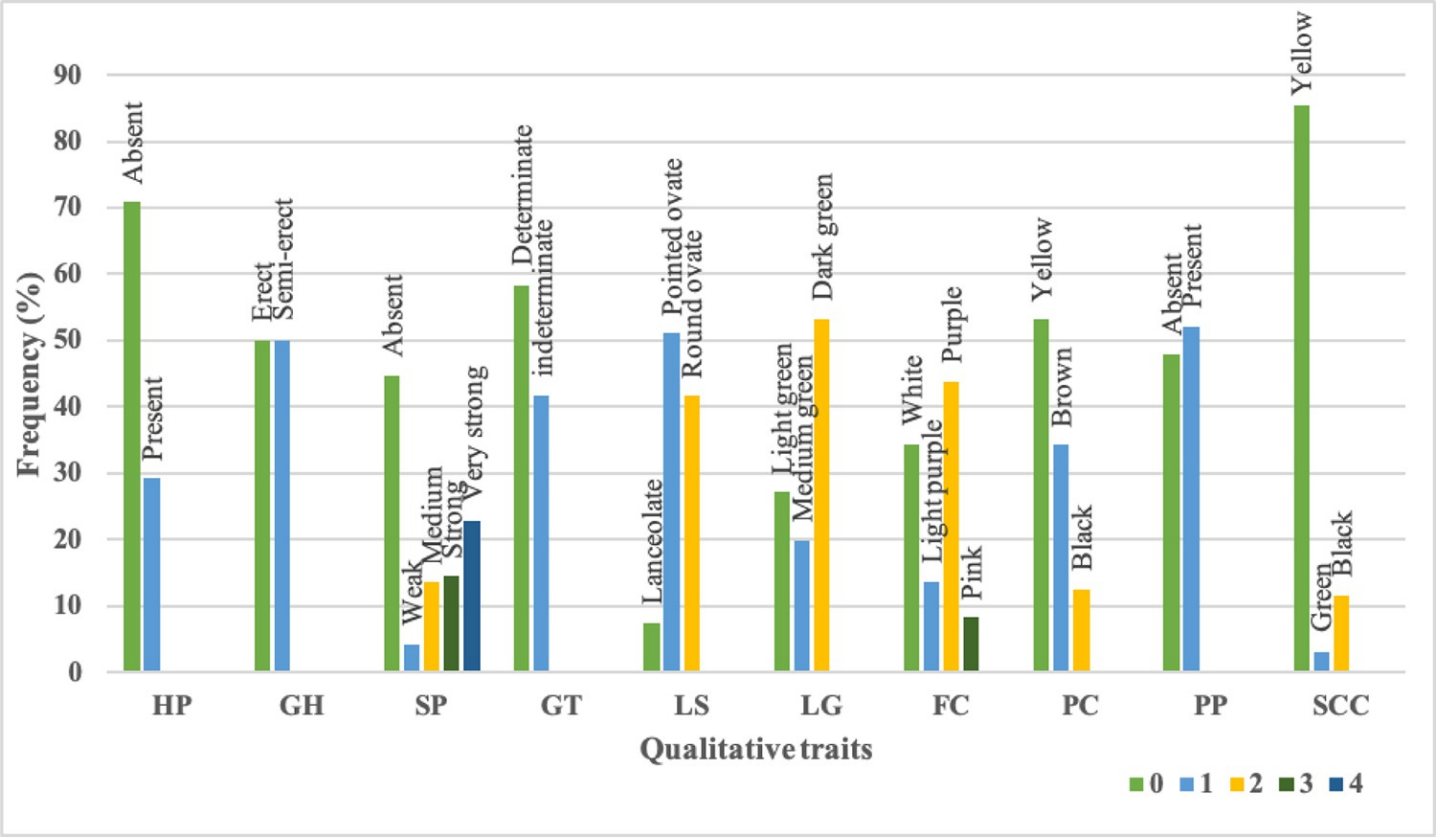
Number of genotypes for each class in different qualitative traits. (**HP**: Hypocotyl anthocyanin pigmentation (0: Absent; 1: Present), **GH**: Growth habit (0: Erect; 1: Semi-erect), **SP**: Stem pubescence (0: Absent; 1: Weak; 2: Medium; 3: Strong; 4: Strong), **GT**: Growth type (0: Determinate; 1: Indeterminate), **LS**: Leaf shape (0: Lanceolate; 1: Pointed ovate; 2: Round ovate), **LG**: Leaf greenness (0: Light green; 1: Medium green; 2: Dark green), **FC**: Flower colour (0: White; 1: Light purple; 2: Purple; 3: Pink), **PC**: Pod colour (0: Yellow; 1: Brown; 2: Black), **PP**: Pod pubescence (0: Absent; 1: Present) and **SCC**: Seed coat colour (0: Yellow; 1: Green; 2: Black).

The variation for ten qualitative traits (HP, GH, GT, FC, LS, LG, SP, PP, PC and SCC) were recorded. The frequency distribution of these traits among 96 soybean genotypes are presented on Fig 7. Most of the genotypes showed yellow seed colour (85.42%) and absence of hypocotyl pigmentation (70.83%). Majority of the genotypes showed determinate growth type (58.33%), pod pubescence (52.08%), pointed ovate leaf shape (51.04%), dark green leaves (53.13%) and yellow pod colour (53.13%). The flower colour ranged from white (34.38%) to pink (8.33%) and most of the genotypes had purple flower (43.75%). Most of the genotypes showed absence of stem pubescence (44.79%) followed by very strong pubescence (22.92%).

**Fig 8:**
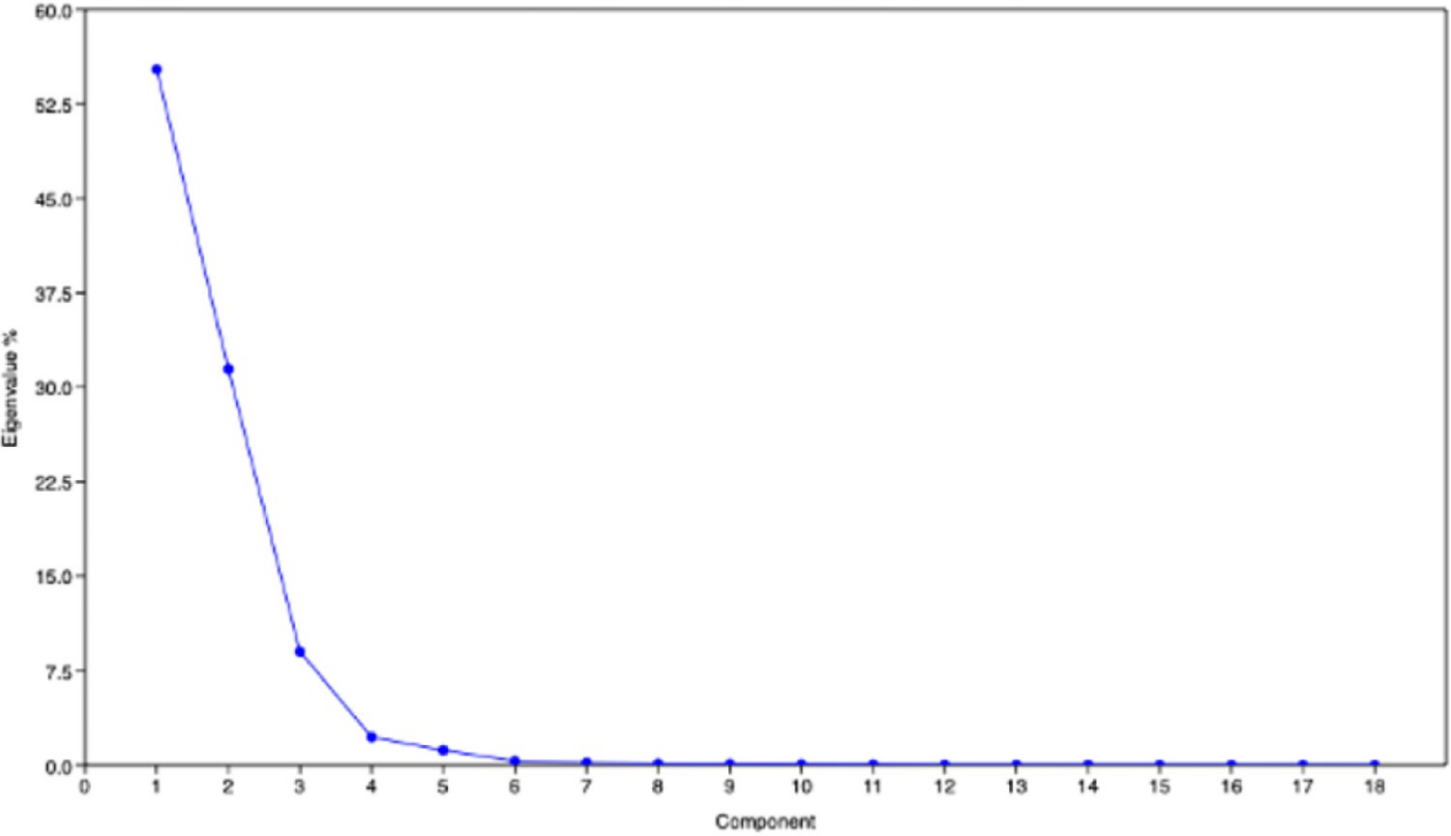
Scree plot of different principal components in PCA using quantitative and qualitative traits.

**Fig 9:**
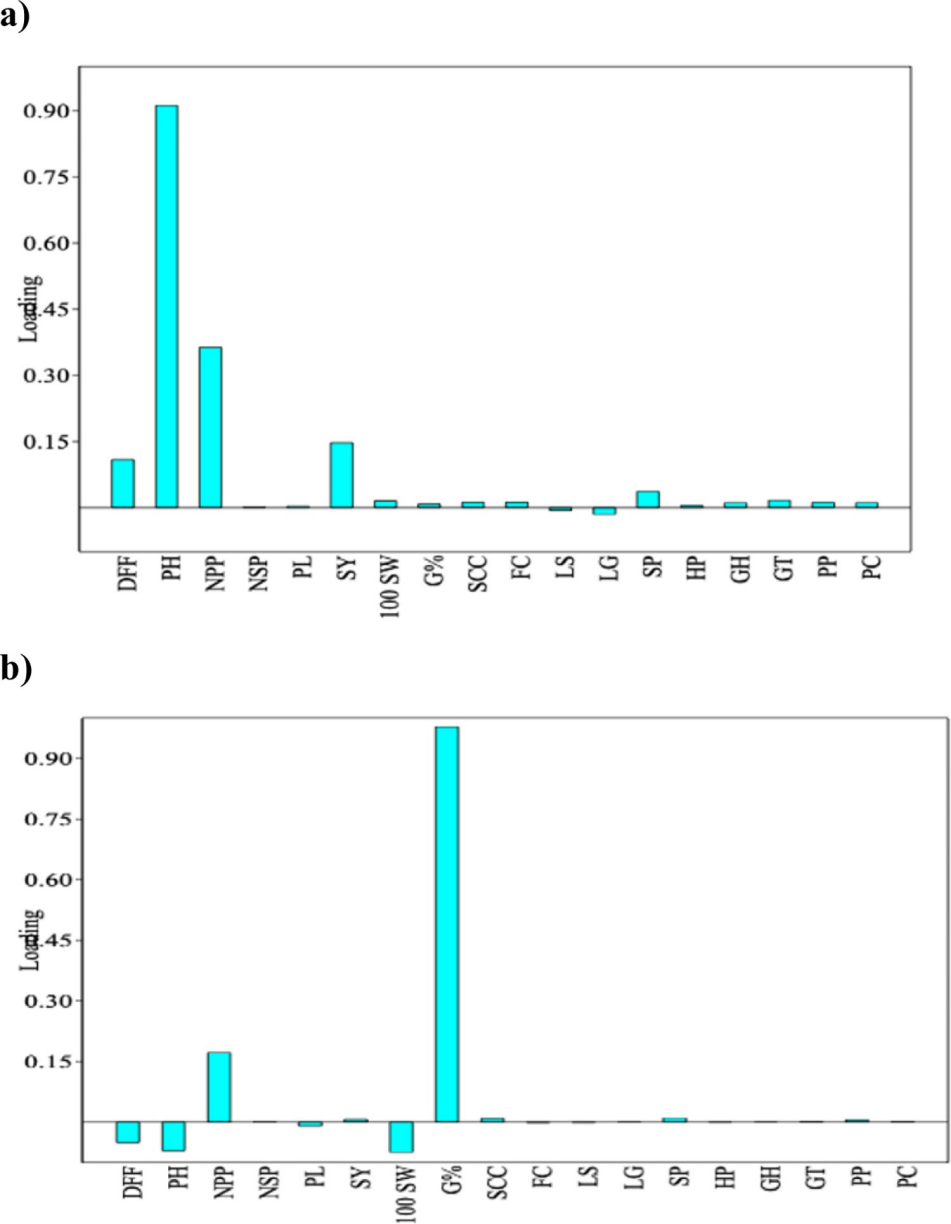
Contribution of quantitative and qualitative traits towards PC 1 (a) and PC 2 (b). DFF: Days to flowering; PH: Plant height; NPP: Number of pods per plant; NSP: Number of seeds per pod; PL: Pod length; SY: Seed yield; 100SW: 100 Seed weight; G%: Seed longevity; SCC: Seed coat colour; FC: Flower colour; LS: Leaf shape; LG: Leaf greenness; SP: Stem pubescence; HP: Hypocotyl anthocyanin pigmentation; GH: Growth habit; GT: Growth type; PP: Pod pubescence; PC: Pod colour.

The PCA was performed using eight quantitative and ten qualitative traits to estimate Eigenvalues and principal components based on trait loadings which are used as covariates for the population structure. Seven components had more than one Eigenvalue and together contribute 99.595 percent variance (Table 6). Principal component 1 (PC 1) and principal component 2 (PC 2) were found to have higher Eigenvalues (Fig 8) with a cumulative percent variance of 86.68 (Table 6). The PC 1 was highly correlated with PH (91.20%) followed by NPP (36.30%) and SY (14.73%) (Fig 9(A)). While, PC 2 was highly associated with G% (97.83%) and NPP (17.14%) (Fig 9(B)). Hence these traits, *i*.*e*., PH, G%, NPP and SY can be used for the determination of genotypes dissimilarity and for grouping the genotypes. Interestingly, PC2 which was associated with seed longevity was negatively associated with 100SW.

**Table 6:** Principal components with its eigenvalues and percentage variances towards the total population variance in PCA using quantitative and qualitative traits.

Principal component	Eigenvalue	% Variance
1	415.14	55.24
2	236.27	31.44
3	67.68	9.06
4	16.73	2.22
5	8.85	1.17
6	2.40	0.32
7	1.36	0.18
8	0.74	0.09
9	0.57	0.07
10	0.45	0.06
11	0.34	0.04
12	0.22	0.03
13	0.19	0.02
14	0.17	0.02
15	0.13	0.02
16	0.11	0.01
17	0.07	0.01
18	0.02	0.002

**Fig 10:**
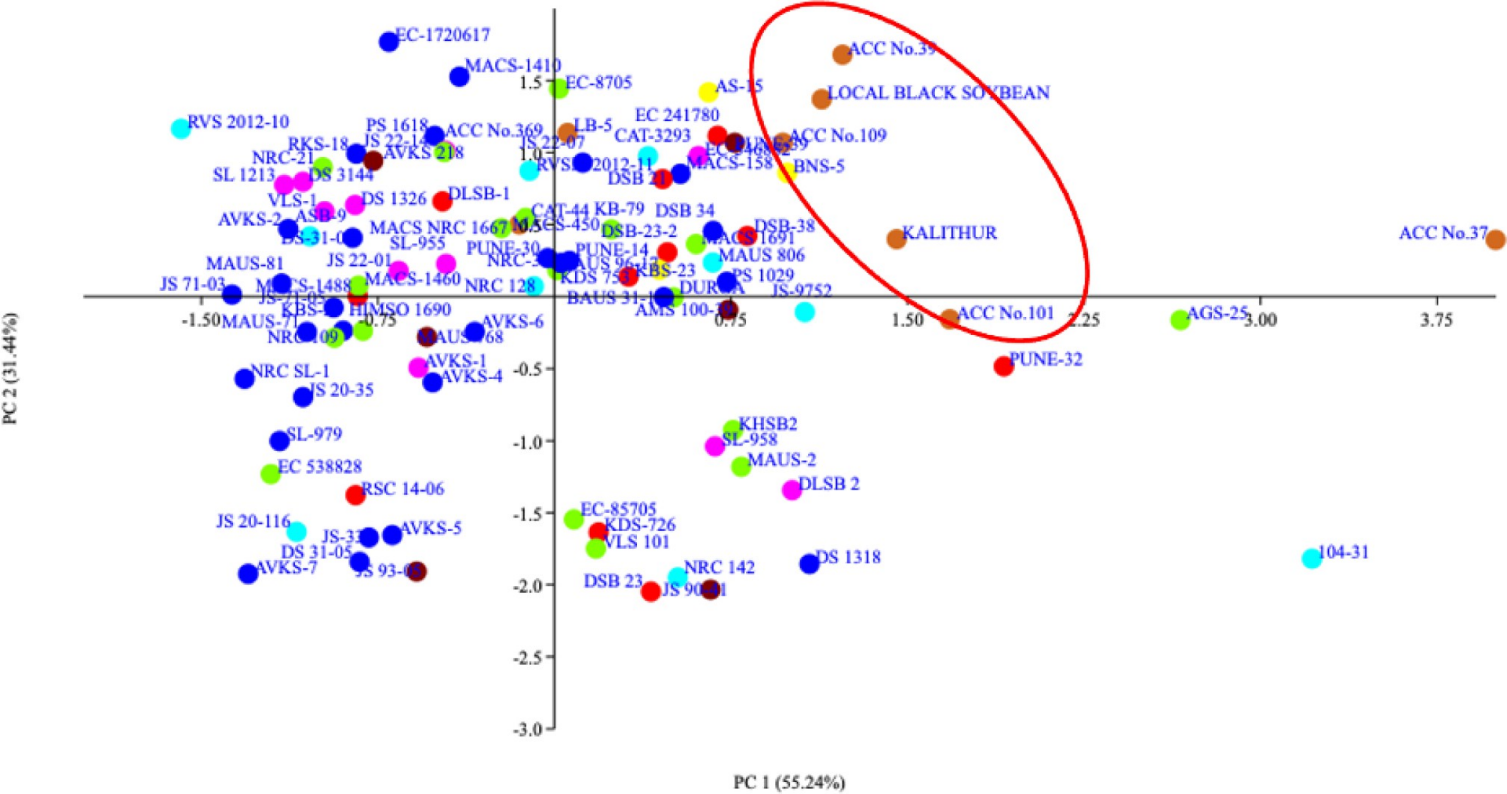
Scatter plot of genotypes based on eigenvalues of components in PC 1 and PC 2. (First quadrant: genotypes with high seed longevity, tall plant height and small seed size (circle consists of black seed coat colour genotypes from C7 sub-population of STRUCTURE analysis using SNP markers); Second quadrant: genotypes with high seed longevity, short plant height and small seed size; Third quadrant: genotypes with low seed longevity, short plant height and bigger seed size; Forth quadrant: genotypes with low seed longevity, tall plant height and bigger seed size).

Based on the score of the genotypes and loading of the traits at PC 1 and PC 2, a scatter plot was made with an unambiguous pattern of genotype combinations on the factor plane (Fig 10). The genotypes with higher G% and smaller seed size with tall PH, and high NPP were placed in the first quadrant, which includes, majority of the black seed coat colour genotypes (ACC Nos. 37, 39 & 109, Local black soybean, LB-5 and Kalitur) from C7 sub-population of STRUCTURE using SNP markers. Second quadrant contained a close cluster having 29 genotypes including three black seed coat colour genotypes with high G% and small seed size with short PH. Nineteen genotypes with low G%, large seed size and short PH were placed in to third quadrant of the scatter plot. The fourth quadrant contained 16 genotypes along with tall PH with low G%.

**Fig 11:**
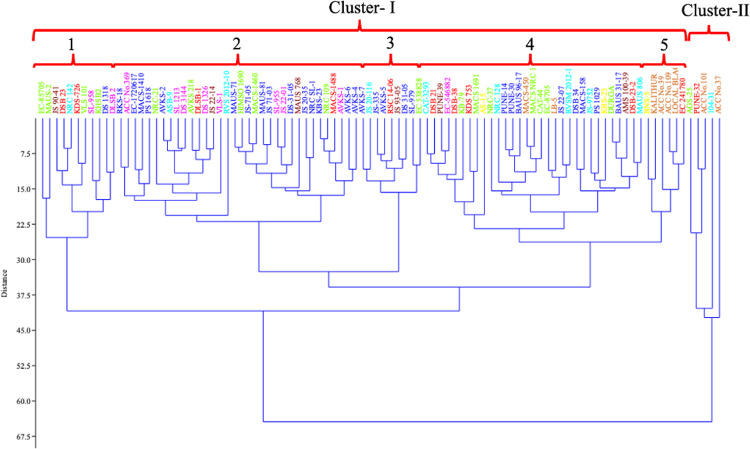
UPGMA based hierarchical clustering of genotypes using quantitative and qualitative traits.

The UPGMA based hierarchical clustering of the genotypes using quantitative and qualitative traits divided the genotypes in to two major clusters (Fig 11). The cluster-I consisted of 89 genotypes and cluster-II consisted of five genotypes. The cluster-I was sub-clustered in to 5 subclusters. The subcluster-5 had genotypes with highest G% (57.66%) and lowest seed size (9.78 gram) (Table 7). Four out of six genotypes in this subcluster were with black seed coat colour. On the contrary the subcluster-1 of cluster-I with 11 genotypes had least G% (17.14%) and highest seed weight (16.76 gram). None of the genotypes had black seed coat colour in this subcluster.

**Table 7:** Number of genotypes, seed longevity (G%) and 100 seed weight (100SW) of clusters formed in hierarchical clustering using phenotypic traits.

Cluster	Sub-cluster	Number of genotypes	Seed longevity (G%)	100 Seed weight (100SW)
**Cluster-I**	1	11	17.14	16.76
	2	35	46.13	12.47
	3	9	17.22	14.73
	4	32	47.94	14.41
	5	6	57.66	9.78
**Cluster-II**		5	35.83	13.56

## Discussion

This study focuses on the diversity and population structure analysis of 96 soybean genotypes assembled to evaluate the seed longevity as well as its association with seed size and seed coat colour. To the best of our knowledge this is the first attempt to assemble the population and understand the genetic population structure to study the seed longevity in soybean. Seed longevity in soybean is a pressing concern since it has been affecting the seed yield and quality and placing seed production under high risk of economic security [9]. Efforts have been made to study the genetic basis of seed longevity and identify the genotypes with high seed longevity in soybean [15, 80, 81]. In this study, 29,955 polymorphic SNPs, generated by GBS analysis of 96 genotypes, were used for population diversity and structure analysis. Various NGS technologies are in high demand due to its efficiency in generating thousands or millions of sequences simultaneously with low cost [82]. Genotyping by sequencing is a popular NGS technique which facilitates marker discovery and high-density genotyping at low cost [55, 83]. This technique enables us to study population structure and identify QTLs of complex traits in large number of genotypes of various crops [83–86]. The high percentage of phred scores (Q20 and Q30) used in the study and GC content of the sequence data revealed sufficient sequencing quality for further variant calling and genotyping. The Q20 and Q30 scores indicate the correction rate of 99.00% and 99.99% for an error rate of 1 in 100 and 1 in 1000 bases, respectively. The high GC content in the sequence reads represents the less linear read coverage and hence wider genome coverage [87]. Higher sequencing depth and mapping rate obtained in the study explains that the Illumina HiSeq^TM^ 2500 sequencing covered almost entire genome. The TASSEL-GBS (v2) pipeline detected variants in every 25,299 bases and found 37,897 polymorphic SNPs on sequences aligned to the reference genome as well as in contigs. The higher number of transitions over transversions indicate that transition mutations are better tolerated during natural selection in soybean which is common to other plant species [88, 89]. Despite the narrowing genomic variation of soybean due to selection pressure during the domestication process [90, 91] and higher level of repetitive DNA in soybean [92], we identified 29,955 high quality SNPs using stringent filtering criteria, detecting higher number of polymorphic SNPs as compared to previous studies [84, 93, 94]. The variant annotation performed using SnpEff [71] showed that majority of the detected SNPs were modifiers in non-coding regions and only few SNPs in coding region resulting in significant changes like frame shift, deletion of large part of chromosome or the entire exon and formation of stop codon. The non-coding SNPs play a significant role in gene regulation and attracted great interest of geneticist in the past decade [95]. The functional classification of the SNPs showed that majority of the variants result in amino acid changes and 19 SNPs results in formation of stop codons and subsequently produce truncate, incomplete or non-functional proteins. The missense to silent ratio was 1.53, which is higher than the ratio reported earlier in soybean [91, 96]. The 29,955 SNPs were distributed in all the chromosomes; however, the highest number of SNPs were found in chromosome-18 and lowest number of SNPs were in chromosome-10.

In this study we used SNP, SSR and agro-morphological traits to analyse the population structure and genetic diversity of 96 soybean genotypes. The Bayesian-model based structuring using SNP markers could effectively identify clusters with higher seed longevity associated with seed coat colour and seed size which were subsequently validated by UPGMA and PCA based on qualitative and quantitative traits. This model has been extensively used to study the optimum population number in various crop species to study the population structure analysis [52, 97–99]. Bayesian-model based population structure analysis yielded eight distinct sub-populations. The average genetic distance and fixation index of the eight sub-populations revealed large genetic divergence existing in the genotypes used for the study. The C7 sub-population with majority of the black seed coat colour genotypes (7 out of 8) had highest seed longevity, plant height and smaller seed size as compared to other sub-populations. The black genotypes with small seed size in sub-population C7 are the valuable resource for seed longevity supporting the earlier results [16, 17, 26].

Both STRUCTURE and UPGMA clustering using SNPs showed that black colour soybean genotypes with small seed size were separated from other soybean genotypes. The UPGMA clustering based phylogenetic tree showed clear grouping of genotypes similar to STRUCTURE analysis. All the genotypes in cluster-II in UPGMA were from C1 and all the 29 genotypes from C3 were grouped in cluster-III. A similar clustering was also formed in SSR based UPGMA clustering with the same 7 black seed coat colour genotypes in a separate cluster as in SNP based UPGMA clustering. Non-parametric population structure analysis using pairwise dissimilarity index with SSR and SNP markers has been widely followed invariably in population genetic studies of cowpea, pigeon pea and soybean [52, 99, 100]. Our study was aimed at characterizing the level of polymorphism in a population assembled for seed longevity based on both SSR and SNP markers, and the study proved that both the marker systems were informative and provided complementary data that helped to describe the population in terms of seed longevity. The SSR and SNP marker system differ for their genomic distribution and basis of polymorphism as well as clustering of genotypes in soybean [54]. Singh et al. [101] and Courtois et al. [102] observed different patterns of cluster using SSR and SNP markers in rice genotypes and reported that the dissimilar grouping of genotypes is obvious irrespective of the markers under investigation. In the present study, we have used only a limited number of SSR markers for clear differentiation of genotypes. Comparatively, SNPs are the marker of choice for population structure analysis and plant breeding [103].

The genotypic or phenotypic information alone may not be efficient to capture the genetic diversity and structure analysis in the population. Therefore, we used both genotypic and phenotypic information for clustering the genotypes in the assembled population. To reveal the phenotypic variation, the 96 genotypes were characterized under field conditions for eight quantitative and ten qualitative traits. The main focus of the assembled population is the genetic variation for seed longevity. The genotypes under investigation were highly diverse for the seed longevity tested under accelerated ageing treatment with high coefficient of variability. Seed longevity assessment using accelerated ageing method was found to be beneficial as compared to natural ageing since it gives a quick and reliable estimate of longevity in soybean [15, 81, 104] and in other crops like chickpea, black gram and common bean [105–107]. We observed a wide variability among the genotypes for seed longevity. The ACC No. 39, EC 1720617, MACS 1410, Local black soybean, ACC No. 369, LB-5, Kalitur, EC 8705 and PS 1618 showed significantly higher seed longevity after the ageing treatment. Five out of 11 black seed coat colour genotypes had more than 70 percent germination after accelerated ageing treatment, indicating higher seed longevity of the black seeded genotypes. All the genotypes with lower seed longevity had yellow seed coat colour. The seed size of the genotypes ranged from 6.60 to 20.80 grams and in general, the black seeded genotypes had smaller seed size except VLS-1. This suggests that substantial variation exists in the assembled population for mapping of QTLs responsible for seed longevity. The other quantitative traits, *viz*., SY, PH, NPP and 100SW showed massive genetic variation with high coefficient of variability suggesting heterogeneity in our assembled population. High level of variability has been reported for plant height [108], seed size [109] and other traits [6, 110, 111] in soybean. These morphological traits are of vital importance as they are linked to yield directly [112]. The qualitative features of the genotypes estimated following DUS guidelines [63] grouped the genotypes in to various categories based on their plant growth and seed characteristics. There is a significant variation in all the qualitative traits which was also reported by earlier studies [28, 113].

The PCA is a powerful approach that allows the understanding on the structure of plant population and it makes possible to identify important variables among the assembled genotypes. The Euclidean biplot showed that the distribution of accessions in all the four quadrants. The scatter plot of PC 1 and PC 2 made using 96 genotypes gave various close clusters in factor planes with majority of the black seed coat colour genotypes *viz*., Local black soybean, Kalitur, ACC Nos. 39, 109, 101 and 37 in the first quadrant that harbour the genotypes with higher seed longevity, plant height and smaller seed size. Interestingly, these six black seed coat colour genotypes were the same genotypes which was clustered together in molecular clustering using SSR and SNP markers as well as SNP based population structure analysis. These genotypes can be identified as genotypes with high seed longevity and grouped together in both molecular and morphological traits based analysis. To date, PCA using morphological and molecular information has been successfully employed to prove the diversity and phylogenetic relationship of genotypes and to select genotypes for breeding [114]. The quantitative and qualitative traits based PCA revealed two main principal components which together explains 86.68 percentage variance. The seed longevity was the most effective trait for PC 2. These results indicate that PH, seed longevity and NPP could be suitable candidate traits to investigate morphogenic variation related to seed longevity in future studies. We also found that seed longevity in the black seed coat colour genotypes showed positive correlation with plant height and number of pods, and negative correlation with seed size. The UPGMA hierarchical clustering using morphological traits revealed two major clusters with 91 genotypes in cluster-I and the remaining 5 genotypes in cluster-II. The genotypes with higher longevity has been considered as an important objective in this study. The cluster analysis of morphological traits illustrate that the subcluster-5 in cluster-I had 6 genotypes with high seed longevity after accelerated ageing treatment. Concomitantly, this group recorded least 100 seed weight suggesting small seed size of the genotypes. The subcluster-1 in cluster-I had 11 genotypes with the lowest seed longevity and largest seed size. The relationship observed between seed coat colour, seed longevity and seed size are in line with the findings of Hosamani et al. [17]. The ability of a seed to sustain its viability during dry seed storage highly varies with the genetic makeup of the seed [115, 116].

The results of STRUCTURE, PCA and UPGMA cluster analysis showed high degree of similarity and provided complementary data that helped to identify genotypes with high seed longevity. We observed that six black seed coat colour genotypes *viz*., local black soybean, Kalitur, ACC Nos. 39, 109, 101 and 37 with high seed longevity and small seed size clustered together in both molecular and morphological distance-based grouping and model-based structuring. The negative correlation between seed longevity in black seed coat colour genotypes and seed size were in agreement with the previous results [12, 14, 16, 17, 104]. High rate of lipid peroxidation and the production of reactive oxygen species are the most reported reasons for low seed longevity in soybean due to its high oil content [20, 117, 118] along with its seed coat composition [119]. Seed coat of black seeded soybean genotypes have high content of hemicellulose [120] which enhances the seed impermeability [121]. High concentration of calcium in the seed coat [122] with lesser gap between testa and cotyledon, a few pores on the seed coat surface, higher lignin content [12] and greater number of secondary metabolites such as anthocyanin and polyphenols [123, 124] are helpful in scavenging the reactive oxygen species created during deterioration [125]. Together with the health benefits of black seed coat colour genotypes [6, 123], higher longevity of the seeds during dry storage makes the black seed coat colour genotypes with enormous utility in soybean crop improvement programme.

## Supporting information

S1 Fig Bar plot of K = 6 using SNP markers. (DOCX)

S1 Table List of soybean genotypes used for the present study and their codes in SNP-UPGMA cluster. (DOCX)

S2 Table List of SSR primers used for the study, their sequence with annealing temperature. (DOCX)

S3 Table Statistics of the GBS-sequencing data. (DOCX)

S4 Table Statistics of mapping rate, depth and coverage. (DOCX)

S5 Table Number and percentage of discovered SNPs in each chromosome out of 26769 chromosome aligned SNPs. (DOCX)

S6 Table Fixation index, expected heterozygosity and number of genotypes in six sub groups of soybean genotypes derived from SNP population structure analysis. (DOCX)
