# Supplementary figures and images for: Population structure and genetic diversity characterization of soybean for seed longevity
Source: PLoS One. 2022 Dec 6;17(12):e0278631. doi: 10.1371/journal.pone.0278631 (PMC9725150; doi:10.1371/journal.pone.0278631)

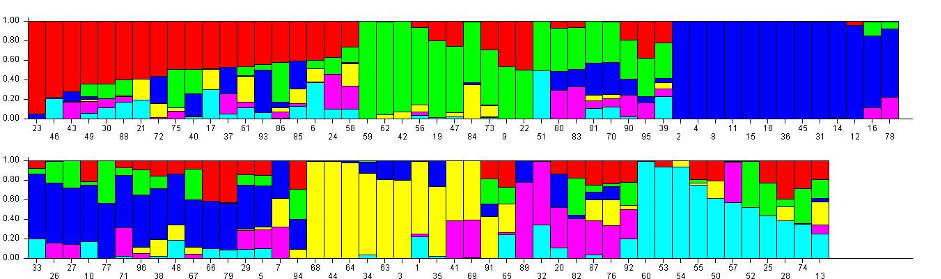


**S1 Fig. Bar plot of K=6 using SNP markers**

Supplement: S1 Fig — (DOCX) [file pone.0278631.s001.docx]
