# Supplementary material for: Population structure and genetic diversity characterization of soybean for seed longevity
Source: PLoS One. 2022 Dec 6;17(12):e0278631. doi: 10.1371/journal.pone.0278631 (PMC9725150; doi:10.1371/journal.pone.0278631)
Supplement: S1 Table — (DOCX) [file pone.0278631.s002.docx]

**S1 table. List of soybean genotypes used for the present study and their codes in SNP-UPGMA cluster**

| Genotype number | Genotypes | Seed coat colour | SNP-UPGMA cluster code |
| --- | --- | --- | --- |
| 1 | EC 241780 | Yellow | S1 |
| 2 | MAUS-81 | Yellow | S10 |
| 3 | DSB-23-2 | Yellow | S11 |
| 4 | AVKS-6 | Yellow | S12 |
| 5 | PUNE-14 | Black | S13 |
| 6 | EC-8705 | Yellow | S14 |
| 7 | DSB 34 | Yellow | S15 |
| 8 | AVKS-7 | Yellow | S16 |
| 9 | CAT-3293 | Yellow | S17 |
| 10 | MACS-158 | Yellow | S18 |
| 11 | RKS-18 | Yellow | S19 |
| 12 | SL-979 | Yellow | S2 |
| 13 | BNS-5 | Green | S20 |
| 14 | JS-335 | Yellow | S21 |
| 15 | MAUS-71 | Yellow | S22 |
| 16 | JS 20-35 | Yellow | S23 |
| 17 | AGS-25 | Yellow | S24 |
| 18 | KBS-23 | Yellow | S25 |
| 19 | SL-955 | Yellow | S26 |
| 20 | PUNE-39 | Yellow | S27 |
| 21 | NRC-37 | Yellow | S28 |
| 22 | JS-9752 | Yellow | S29 |
| 23 | NRC-21 | Yellow | S3 |
| 24 | 104-31 | Green | S30 |
| 25 | MACS-450 | Yellow | S31 |
| 26 | JS-71-05 | Yellow | S32 |
| 27 | JS 71-03 | Yellow | S33 |
| 28 | EC-85705 | Yellow | S34 |
| 29 | PUNE-30 | Black | S35 |
| 30 | MAUS-2 | Yellow | S36 |
| 31 | AVKS-4 | Yellow | S37 |
| 32 | JS 90-41 | Green | S38 |
| 33 | EC-1720617 | Yellow | S39 |
| 34 | PUNE-32 | Yellow | S4 |
| 35 | MACS-1488 | Yellow | S40 |
| 36 | AVKS-5 | Yellow | S41 |
| 37 | MACS-1460 | Yellow | S42 |
| 38 | PS 1618 | Yellow | S43 |
| 39 | DURGA | Yellow | S44 |
| 40 | JS 20-116 | Yellow | S45 |
| 41 | KDS-726 | Yellow | S46 |
| 42 | SL-958 | Yellow | S47 |
| 43 | CAT-44 | Yellow | S48 |
| 44 | RSC 14-06 | Yellow | S49 |
| 45 | AVKS-2 | Yellow | S5 |
| 46 | KHSB2 | Yellow | S50 |
| 47 | AVKS-1 | Yellow | S51 |
| 48 | MACS-1410 | Yellow | S52 |
| 49 | KB-79 | Yellow | S53 |
| 50 | ACC No.37 | Black | S54 |
| 51 | ACC No.369 | Black | S55 |
| 52 | KALITHUR | Black | S56 |
| 53 | ACC No.39 | Black | S57 |
| 54 | ACC No.109 | Black | S58 |
| 55 | ACC No.101 | Black | S59 |
| 56 | EC-546882 | Yellow | S6 |
| 57 | LB-5 | Black | S60 |
| 58 | EC 538828 | Yellow | S61 |
| 59 | VLS-1 | Black | S62 |
| 60 | LOCAL BLACK SOYBEAN | Black | S63 |
| 61 | HIMSO 1690 | Yellow | S64 |
| 62 | SL 1213 | Yellow | S65 |
| 63 | DSB 23 | Yellow | S66 |
| 64 | DSB 21 | Yellow | S67 |
| 65 | AVKS 218 | Yellow | S68 |
| 66 | DS 1318 | Yellow | S69 |
| 67 | DS-31-05 | Yellow | S7 |
| 68 | DSB-38 | Yellow | S70 |
| 69 | KDS 753 | Yellow | S71 |
| 70 | DS 1326 | Yellow | S72 |
| 71 | JS 22-07 | Yellow | S73 |
| 72 | KBS-21 | Yellow | S74 |
| 73 | JS 22-01 | Yellow | S75 |
| 74 | MACS NRC 1667 | Yellow | S76 |
| 75 | NRC 142 | Yellow | S77 |
| 76 | AMS 100-39 | Yellow | S78 |
| 77 | NRC SL-1 | Yellow | S79 |
| 78 | PS 1029 | Yellow | S8 |
| 79 | BAUS 96-17 | Yellow | S80 |
| 80 | ASB-9 | Yellow | S81 |
| 81 | DS 3144 | Yellow | S82 |
| 82 | RVS 2012-10 | Yellow | S83 |
| 83 | JS 22-14 | Yellow | S84 |
| 84 | DLSB 2 | Yellow | S85 |
| 85 | AS-15 | Yellow | S86 |
| 86 | VLS 101 | Yellow | S87 |
| 87 | MAUS 768 | Yellow | S88 |
| 88 | MACS 1691 | Yellow | S89 |
| 89 | JS 93-05 | Yellow | S9 |
| 90 | MAUS 806 | Yellow | S90 |
| 91 | DLSB-1 | Yellow | S91 |
| 92 | NRC 109 | Yellow | S92 |
| 93 | BAUS 31-17 | Yellow | S93 |
| 94 | NRC 128 | Yellow | S94 |
| 95 | RVSM 2012-11 | Yellow | S95 |
| 96 | DS 3-05 | Yellow | S96 |
