# Supplementary material for: Population structure and genetic diversity characterization of soybean for seed longevity
Source: PLoS One. 2022 Dec 6;17(12):e0278631. doi: 10.1371/journal.pone.0278631 (PMC9725150; doi:10.1371/journal.pone.0278631)
Supplement: S2 Table — (DOCX) [file pone.0278631.s003.docx]

**S2 table. List of SSR primers used for the study, their sequence with annealing temperature**

| Primer number | Primer name | Motif | Forward sequence | Reverse sequence | Annealing temperature (^0^C) |
| --- | --- | --- | --- | --- | --- |
| **BSOY18** | BARCSOYSSR_01_0211 | (AG)16 | CCTTCTATCCGCAAAACCCT | ATATCACTCCCTCCCCAACC | 55.5 |
| **BSOY20** | BARCSOYSSR_01_1484 | (TC)18 | CTTCTCTCAGCACCCTCCAC | AACCCTTCTTCCACTTCCGT | 54.5 |
| **BSOY28** | BARCSOYSSR_04_0414 | (CT)17 | CCATTCTACAATCATGCCCC | AGAAGCTGGCTAAGATGGCA | 57.5 |
| **BSOY34** | BARCSOYSSR_08_1190 | (TC)16 | AATCATCTCCCAAGGAGTGC | TGGCAAACTTTGGTCATTCA | 55.0 |
| **BSOY36** | BARCSOYSSR_08_1346 | (GT)16 | TATGTGTGTGTGTGTGGGGG | GTGCTGGCTTTTCAACCAAA | 57.5 |
| **BSOY43** | BARCSOYSSR_09_0179 | (AG)20 | ACCAATTGCCAACAACACAA | GTCCTACAAGGCCCACAAAA | 55.0 |
| **BSOY47** | BARCSOYSSR_10_0267 | (CT)17 | ACATCGTTCTCCCTCTCCCT | CCTTCTTCCCAGAACCATCA | 57.5 |
| **BSOY45** | BARCSOYSSR_11_1391 | (TA)20 | CTTTTGGCACCACAAATTCC | TGGGCTCATTACGAGCATCT | 51.5 |
| **BSOY1** | BARCSOYSSR_12_0930 | (GA)18 | CCACCACTCATCAACACCTG | CGTCAAGGTTCCCTTACCCT | 54.5 |
| **BSOY4** | BARCSOYSSR_14_1271 | (CT)16 | AAGGAAGGAAAACCCATGCT | GGGACCACAGCGTTGAATTA | 51.5 |
| **BSOY6** | BARCSOYSSR_15_1563 | (TC)18 | GCGCAAGCACTGAATGTCA | GCGTCACTAACACCTATAACA | 51.5 |
| **BSOY19** | BARCSOYSSR_18_1942 | (TC)19 | CTCCTCATGCTTGGCAAAAT | ATGAGAACGCTGAAAAGGGA | 55.0 |
| **BSOY23** | BARCSOYSSR_19_0884 | (AG)16 | CCAAGTAGCGGGGTTACAAA | GACCATAGTAGCGAGGGCTG | 54.5 |
| **BSOY29** | BARCSOYSSR_19_1324 | (TC)16 | TCTCTTTTCACGGTGGCTTC | AAGGAGTGGTTGTGGGTTTG | 57.5 |
| **SATT453** | BARCSOYSSR_11_1468 | (ATT)14 | GCGGAAAAAAAACAATAAACAACA | TAGTGGGGAAGGGAAGTTACC | 55.5 |
