# Supplementary material for: Population structure and genetic diversity characterization of soybean for seed longevity
Source: PLoS One. 2022 Dec 6;17(12):e0278631. doi: 10.1371/journal.pone.0278631 (PMC9725150; doi:10.1371/journal.pone.0278631)
Supplement: S3 Table — (DOCX) [file pone.0278631.s004.docx]

**S3 table. Statistics of the GBS-sequencing data**

| Sample | Raw bases(bp) | Clean bases(bp) | Effective rate(%) | Error rate(%) | Q20(%) | Q30(%) | GC content(%) |  |
| --- | --- | --- | --- | --- | --- | --- | --- | --- |
| EC 241780 | | 326959200 | 326934720 | 99.99 | 0.04 | 96.17 | 89.31 | 35.77 |
| MAUS-81 | | 270052992 | 270041472 | 100 | 0.04 | 96.88 | 90.92 | 36.7 |
| DSB-23-2 | | 286365024 | 286354368 | 100 | 0.04 | 96.6 | 90.26 | 36.7 |
| AVKS-6 | | 253006848 | 252994176 | 99.99 | 0.04 | 95.8 | 88.41 | 35.84 |
| PUNE-14 | | 351047520 | 351028512 | 99.99 | 0.05 | 95.65 | 88.06 | 36.83 |
| EC-8705 | | 340666848 | 340647840 | 99.99 | 0.05 | 95.44 | 87.53 | 37.12 |
| DSB 34 | | 322861248 | 322838784 | 99.99 | 0.05 | 95.43 | 87.47 | 37.16 |
| AVKS-7 | | 322263648 | 322248096 | 100 | 0.04 | 96.27 | 89.47 | 36.79 |
| CAT-3293 | | 347093856 | 347078880 | 100 | 0.04 | 96.92 | 91.17 | 36.74 |
| MACS-158 | | 329919840 | 329903136 | 99.99 | 0.04 | 96.3 | 89.64 | 35.76 |
| RKS-18 | | 307505088 | 307488096 | 99.99 | 0.04 | 96.45 | 89.96 | 36.8 |
| SL-979 | | 261409536 | 261388800 | 99.99 | 0.04 | 96.31 | 89.53 | 36.92 |
| BNS-5 | | 362079936 | 362065536 | 100 | 0.04 | 97.15 | 91.69 | 36.56 |
| JS-335 | | 334624608 | 334602720 | 99.99 | 0.04 | 96.77 | 90.69 | 36.72 |
| MAUS-71 | | 261754560 | 261745056 | 100 | 0.05 | 95 | 86.6 | 36.2 |
| JS 20-35 | | 353607552 | 353583648 | 99.99 | 0.05 | 95.62 | 88.05 | 37.12 |
| AGS-25 | | 379902816 | 379880928 | 99.99 | 0.04 | 96.12 | 89.19 | 36.88 |
| KBS-23 | | 303704640 | 303691968 | 100 | 0.05 | 94.94 | 86.52 | 37.25 |
| SL-955 | | 220360032 | 220350240 | 100 | 0.04 | 97.13 | 91.59 | 36.68 |
| PUNE-39 | | 281725920 | 281707776 | 99.99 | 0.04 | 97.38 | 92.17 | 36.63 |
| NRC-37 | | 311569920 | 311548896 | 99.99 | 0.04 | 96.16 | 89.19 | 36.85 |
| JS-9752 | | 331382016 | 331362720 | 99.99 | 0.04 | 96.42 | 89.9 | 37.02 |
| NRC-21 | | 275132736 | 275124096 | 100 | 0.05 | 95.7 | 88.27 | 36.58 |
| 104-31 | | 301161024 | 301150080 | 100 | 0.05 | 95.42 | 87.54 | 37.11 |
| MACS-450 | | 328833504 | 328811904 | 99.99 | 0.05 | 94.94 | 86.5 | 36.94 |
| JS-71-05 | | 328464288 | 328443264 | 99.99 | 0.04 | 96.36 | 89.67 | 36.58 |
| JS 71-03 | | 339404544 | 339387840 | 100 | 0.05 | 95.5 | 87.75 | 37.2 |
| EC-85705 | | 332911584 | 293157216 | 88.06 | 0.06 | 94.59 | 85 | 37.27 |
| PUNE-30 | | 218668320 | 218659392 | 100 | 0.04 | 96.3 | 89.58 | 35.66 |
| MAUS-2 | | 333422496 | 333400032 | 99.99 | 0.04 | 96.46 | 89.92 | 36.72 |
| AVKS-4 | | 192811680 | 192802464 | 100 | 0.04 | 95.86 | 88.69 | 35.25 |
| JS 90-41 | | 286217856 | 286207488 | 100 | 0.04 | 95.87 | 88.69 | 36.56 |
| EC-1720617 | | 290071872 | 290048544 | 99.99 | 0.05 | 95.42 | 87.72 | 36.4 |
| PUNE-32 | | 369440352 | 369414432 | 99.99 | 0.04 | 96.48 | 90.08 | 36.3 |
| MACS-1488 | | 292826016 | 292813920 | 100 | 0.04 | 96.22 | 89.6 | 36.27 |
| AVKS-5 | | 333034848 | 333010944 | 99.99 | 0.04 | 96.25 | 89.61 | 36.59 |
| MACS-1460 | | 254945376 | 254932992 | 100 | 0.04 | 95.91 | 88.79 | 36.78 |
| PS 1618 | | 276449760 | 276428736 | 99.99 | 0.05 | 94.78 | 86.27 | 36.79 |
| DURGA | | 260234784 | 260220960 | 99.99 | 0.04 | 96.86 | 91.09 | 36.44 |
| JS 20-116 | | 241510752 | 241498944 | 100 | 0.04 | 96.16 | 89.37 | 36.73 |
| KDS-726 | | 229787424 | 229772448 | 99.99 | 0.04 | 96.56 | 90.36 | 36.66 |
| SL-958 | | 288335808 | 288314208 | 99.99 | 0.04 | 96.17 | 89.47 | 36.56 |
| CAT-44 | | 311040576 | 311026752 | 100 | 0.05 | 95.48 | 87.83 | 36.06 |
| RSC 14-06 | | 333588384 | 333570528 | 99.99 | 0.05 | 95.28 | 87.43 | 36.77 |
| AVKS-2 | | 296472672 | 296459712 | 100 | 0.04 | 96.55 | 90.17 | 36.73 |
| KHSB2 | | 238397184 | 238381920 | 99.99 | 0.05 | 94.85 | 86.47 | 36.78 |
| AVKS-1 | | 245163168 | 245151072 | 100 | 0.05 | 94.87 | 86.48 | 36.88 |
| MACS-1410 | | 263744928 | 263730816 | 99.99 | 0.04 | 95.9 | 88.78 | 36.58 |
| KB-79 | | 256490496 | 256478400 | 100 | 0.04 | 96.59 | 90.52 | 36.41 |
| ACC No.37 | | 269007552 | 268990560 | 99.99 | 0.04 | 96.13 | 89.38 | 36.67 |
| ACC No.369 | | 240952320 | 240939072 | 99.99 | 0.04 | 96.11 | 89.34 | 36.69 |
| KALITHUR | | 239890752 | 239873472 | 99.99 | 0.04 | 96.91 | 91.21 | 36.52 |
| ACC No.39 | | 221505120 | 221494752 | 100 | 0.04 | 96.55 | 90.28 | 37.3 |
| ACC No.109 | | 233880480 | 233866656 | 99.99 | 0.05 | 94.44 | 85.62 | 35.88 |
| ACC No.101 | | 243093600 | 243077472 | 99.99 | 0.05 | 95.07 | 87.05 | 36.24 |
| EC-546882 | | 291783168 | 291772512 | 100 | 0.04 | 96.17 | 89.28 | 36.7 |
| LB-5 | | 259234560 | 259221024 | 99.99 | 0.04 | 95.75 | 88.48 | 36.55 |
| EC 538828 | | 296155584 | 296132832 | 99.99 | 0.05 | 94.55 | 85.83 | 37.08 |
| VLS-1 | | 288999648 | 288979776 | 99.99 | 0.04 | 96.72 | 90.81 | 36.48 |
| LOCAL BLACK SOYBEAN | | 154124352 | 154115712 | 99.99 | 0.04 | 97.39 | 92.23 | 38.13 |
| HIMSO 1690 | | 266269248 | 266252832 | 99.99 | 0.05 | 95.83 | 88.56 | 36.74 |
| SL 1213 | | 243000288 | 242985600 | 99.99 | 0.04 | 96.06 | 89.16 | 36.8 |
| DSB 23 | | 245996352 | 245979360 | 99.99 | 0.05 | 95.05 | 86.86 | 36.86 |
| DSB 21 | | 242218656 | 242201376 | 99.99 | 0.05 | 94.53 | 85.8 | 36.57 |
| AVKS 218 | | 231608736 | 231592608 | 99.99 | 0.04 | 95.98 | 88.97 | 36.36 |
| DS 1318 | | 301967136 | 301945248 | 99.99 | 0.05 | 95.1 | 86.99 | 36.49 |
| DS-31-05 | | 209967840 | 209955744 | 99.99 | 0.05 | 95.37 | 87.33 | 37.07 |
| DSB-38 | | 287848800 | 240033312 | 83.39 | 0.06 | 94.61 | 85 | 37.24 |
| KDS 753 | | 176238720 | 176229504 | 99.99 | 0.04 | 95.81 | 88.68 | 35.25 |
| DS 1326 | | 276959232 | 276947136 | 100 | 0.04 | 96.11 | 89.21 | 36.31 |
| JS 22-07 | | 304664832 | 304659072 | 100 | 0.04 | 97.45 | 92.28 | 35.88 |
| KBS-21 | | 215044704 | 215038656 | 100 | 0.04 | 97.38 | 92.14 | 36.44 |
| JS 22-01 | | 323375616 | 323375616 | 100 | 0.04 | 97.14 | 91.48 | 36.44 |
| MACS NRC 1667 | | 332220096 | 332220096 | 100 | 0.04 | 97.5 | 92.53 | 35.75 |
| NRC 142 | | 321922368 | 321916320 | 100 | 0.04 | 97.51 | 92.53 | 36.3 |
| AMS 100-39 | | 308658528 | 308658528 | 100 | 0.04 | 97.36 | 92.07 | 36.07 |
| NRC SL-1 | | 280082304 | 280082304 | 100 | 0.04 | 96.74 | 90.42 | 36.48 |
| PS 1029 | | 283998240 | 283983552 | 99.99 | 0.04 | 97.06 | 91.45 | 36.03 |
| BAUS 96-17 | | 310728960 | 310728960 | 100 | 0.04 | 97.94 | 93.62 | 35.76 |
| ASB-9 | | 293633568 | 293631552 | 100 | 0.04 | 97.42 | 92.3 | 36.09 |
| DS 3144 | | 271494720 | 271494720 | 100 | 0.04 | 97.8 | 93.24 | 36.33 |
| RVS 2012-10 | | 307999584 | 307991232 | 100 | 0.04 | 97.57 | 92.66 | 36.11 |
| JS 22-14 | | 315429696 | 315423360 | 100 | 0.04 | 97.11 | 91.49 | 35.88 |
| DLSB 2 | | 329494752 | 329489568 | 100 | 0.04 | 96.95 | 91.01 | 36.22 |
| AS-15 | | 381479040 | 381471552 | 100 | 0.04 | 96.78 | 90.58 | 36.24 |
| VLS 101 | | 326061216 | 326054016 | 100 | 0.04 | 96.81 | 90.62 | 36.53 |
| MAUS 768 | | 346987872 | 346987872 | 100 | 0.04 | 97.42 | 92.19 | 36.17 |
| MACS 1691 | | 279008928 | 279008928 | 100 | 0.04 | 97.87 | 93.47 | 36.27 |
| JS 93-05 | | 306464544 | 306453888 | 100 | 0.04 | 96.25 | 89.48 | 35.88 |
| MAUS 806 | | 352390752 | 352380096 | 100 | 0.04 | 97.53 | 92.52 | 36.18 |
| DLSB-1 | | 352628640 | 352628640 | 100 | 0.04 | 97.44 | 92.33 | 36.05 |
| NRC 109 | | 368177472 | 368172000 | 100 | 0.04 | 98.01 | 93.83 | 35.99 |
| BAUS 31-17 | | 371525184 | 371525184 | 100 | 0.04 | 97.69 | 92.95 | 36.11 |
| NRC 128 | | 323703072 | 323700480 | 100 | 0.04 | 96.49 | 89.9 | 35.54 |
| RVSM 2012-11 | | 337913568 | 337913568 | 100 | 0.04 | 97.06 | 91.27 | 36.79 |
| DS 3-05 | | 344185344 | 344178432 | 100 | 0.04 | 97.31 | 91.95 | 36.28 |
