# Supplementary material for: Population structure and genetic diversity characterization of soybean for seed longevity
Source: PLoS One. 2022 Dec 6;17(12):e0278631. doi: 10.1371/journal.pone.0278631 (PMC9725150; doi:10.1371/journal.pone.0278631)
Supplement: S4 Table — (DOCX) [file pone.0278631.s005.docx]

**S4 table. Statistics of mapping rate, depth and coverage**

| Sample | Clean reads | Mapped reads | Mapping rate(%) | Average depth(X) | Coverage 1X(%) | Coverage 4X(%) |
| --- | --- | --- | --- | --- | --- | --- |
| EC 241780 | 2119738 | 2112366 | 99.65 | 8.43 | 3.54 | 2.05 |
| MAUS-81 | 1759640 | 1752744 | 99.61 | 6.28 | 4 | 1.96 |
| DSB-23-2 | 1858476 | 1849830 | 99.53 | 6.45 | 4.1 | 2.04 |
| AVKS-6 | 1632378 | 1625911 | 99.6 | 7.11 | 3.27 | 1.79 |
| PUNE-14 | 2270372 | 2261974 | 99.63 | 8.22 | 3.85 | 2.12 |
| EC-8705 | 2189480 | 2179791 | 99.56 | 7.46 | 4.15 | 2.18 |
| DSB 34 | 2082688 | 2074084 | 99.59 | 7.08 | 4.19 | 2.15 |
| AVKS-7 | 2098212 | 2089708 | 99.59 | 7.59 | 3.89 | 2.09 |
| CAT-3293 | 2268036 | 2259717 | 99.63 | 7.65 | 4.15 | 2.19 |
| MACS-158 | 2150264 | 2142423 | 99.64 | 8.67 | 3.49 | 2.05 |
| RKS-18 | 2002184 | 1993800 | 99.58 | 7.24 | 3.94 | 2.08 |
| SL-979 | 1643608 | 1636775 | 99.58 | 5.89 | 4 | 1.9 |
| BNS-5 | 2380884 | 2371593 | 99.61 | 8.31 | 4.06 | 2.25 |
| JS-335 | 2177864 | 2169129 | 99.6 | 7.01 | 4.37 | 2.21 |
| MAUS-71 | 1679832 | 1670701 | 99.46 | 7.23 | 3.32 | 1.84 |
| JS 20-35 | 2274222 | 2263918 | 99.55 | 8.1 | 3.94 | 2.14 |
| AGS-25 | 2454304 | 2444603 | 99.6 | 7.87 | 4.41 | 2.34 |
| KBS-23 | 1939818 | 1931693 | 99.58 | 6.91 | 3.98 | 2.02 |
| SL-955 | 1384216 | 1373519 | 99.23 | 5.46 | 3.59 | 1.59 |
| PUNE-39 | 1854574 | 1847327 | 99.61 | 6.76 | 3.9 | 1.99 |
| NRC-37 | 1997944 | 1990523 | 99.63 | 6.7 | 4.17 | 2.06 |
| JS-9752 | 2148030 | 2139661 | 99.61 | 6.84 | 4.45 | 2.21 |
| NRC-21 | 1779142 | 1771926 | 99.59 | 7.47 | 3.35 | 1.85 |
| 104-31 | 1938020 | 1929021 | 99.54 | 7.08 | 3.88 | 2.02 |
| MACS-450 | 2101596 | 2093749 | 99.63 | 7.35 | 4.03 | 2.09 |
| JS-71-05 | 2136024 | 2128500 | 99.65 | 7.49 | 4.04 | 2.13 |
| JS 71-03 | 2186116 | 2177091 | 99.59 | 7.41 | 4.17 | 2.16 |
| EC-85705 | 1895038 | 1886839 | 99.57 | 7.69 | 3.5 | 1.89 |
| PUNE-30 | 1419628 | 1414395 | 99.63 | 6.41 | 3.18 | 1.68 |
| MAUS-2 | 2161976 | 2152723 | 99.57 | 7.48 | 4.09 | 2.15 |
| AVKS-4 | 1199182 | 1194277 | 99.59 | 5.84 | 2.95 | 1.35 |
| JS 90-41 | 1836578 | 1829858 | 99.63 | 6.66 | 3.92 | 1.99 |
| EC-1720617 | 1856570 | 1849328 | 99.61 | 7.44 | 3.54 | 1.97 |
| PUNE-32 | 2399580 | 2391181 | 99.65 | 8.76 | 3.84 | 2.19 |
| MACS-1488 | 1871808 | 1865361 | 99.66 | 6.94 | 3.81 | 1.95 |
| AVKS-5 | 2151490 | 2141275 | 99.53 | 7.28 | 4.17 | 2.19 |
| MACS-1460 | 1633888 | 1627454 | 99.61 | 6.01 | 3.9 | 1.88 |
| PS 1618 | 1742682 | 1734098 | 99.51 | 6.23 | 3.96 | 1.89 |
| DURGA | 1678700 | 1672549 | 99.63 | 6.01 | 3.97 | 1.88 |
| JS 20-116 | 1531430 | 1524713 | 99.56 | 5.64 | 3.91 | 1.81 |
| KDS-726 | 1474642 | 1468654 | 99.59 | 5.08 | 4.18 | 1.79 |
| SL-958 | 1848522 | 1841420 | 99.62 | 6.25 | 4.22 | 2.04 |
| CAT-44 | 1954988 | 1948491 | 99.67 | 7.28 | 3.82 | 1.99 |
| RSC 14-06 | 2121164 | 2113665 | 99.65 | 7.5 | 4.05 | 2.17 |
| AVKS-2 | 1928372 | 1920584 | 99.6 | 7.08 | 3.87 | 2.02 |
| KHSB2 | 1511690 | 1505847 | 99.61 | 6.2 | 3.5 | 1.74 |
| AVKS-1 | 1553112 | 1546637 | 99.58 | 6.1 | 3.66 | 1.78 |
| MACS-1410 | 1693994 | 1687710 | 99.63 | 6.45 | 3.73 | 1.88 |
| KB-79 | 1656118 | 1649736 | 99.61 | 6.29 | 3.74 | 1.85 |
| ACC No.37 | 1731778 | 1725093 | 99.61 | 6.62 | 3.76 | 1.94 |
| ACC No.369 | 1550658 | 1545269 | 99.65 | 5.72 | 3.9 | 1.83 |
| KALITHUR | 1557004 | 1551201 | 99.63 | 5.77 | 3.86 | 1.8 |
| ACC No.39 | 1433020 | 1427083 | 99.59 | 5.4 | 3.82 | 1.71 |
| ACC No.109 | 1458138 | 1452990 | 99.65 | 6.44 | 3.25 | 1.68 |
| ACC No.101 | 1534966 | 1530138 | 99.69 | 6.65 | 3.3 | 1.73 |
| EC-546882 | 1893580 | 1882667 | 99.42 | 7.41 | 3.6 | 1.94 |
| LB-5 | 1657092 | 1651239 | 99.65 | 6.22 | 3.81 | 1.88 |
| EC 538828 | 1850750 | 1843040 | 99.58 | 6.58 | 4.01 | 1.99 |
| VLS-1 | 1874216 | 1867245 | 99.63 | 6.86 | 3.92 | 2.06 |
| LOCAL BLACK SOYBEAN | 1010160 | 1006024 | 99.59 | 4.65 | 3.13 | 1.24 |
| HIMSO 1690 | 1704040 | 1694260 | 99.43 | 5.78 | 4.09 | 1.8 |
| SL 1213 | 1565030 | 1558602 | 99.59 | 5.63 | 3.91 | 1.71 |
| DSB 23 | 1566250 | 1559455 | 99.57 | 5.8 | 3.79 | 1.7 |
| DSB 21 | 1523282 | 1516916 | 99.58 | 5.7 | 3.76 | 1.65 |
| AVKS 218 | 1485678 | 1477059 | 99.42 | 5.95 | 3.52 | 1.63 |
| DS 1318 | 1928236 | 1920800 | 99.61 | 6.99 | 3.74 | 1.89 |
| DS-31-05 | 1272868 | 1266728 | 99.52 | 4.6 | 3.98 | 1.53 |
| DSB-38 | 1547350 | 1542001 | 99.65 | 6.31 | 3.46 | 1.64 |
| KDS 753 | 1125922 | 1121780 | 99.63 | 5.59 | 2.87 | 1.3 |
| DS 1326 | 1777774 | 1749513 | 98.41 | 6.84 | 3.62 | 1.86 |
| JS 22-07 | 1992908 | 1986049 | 99.66 | 6.61 | 4.22 | 2.06 |
| KBS-21 | 1407678 | 1402551 | 99.64 | 4.97 | 4.02 | 1.61 |
| JS 22-01 | 2136030 | 2127360 | 99.59 | 7.48 | 3.95 | 2.12 |
| MACS NRC 1667 | 2194030 | 2187123 | 99.69 | 7.82 | 3.83 | 2.14 |
| NRC 142 | 2122718 | 2115343 | 99.65 | 7.36 | 4 | 2.12 |
| AMS 100-39 | 2035620 | 2027929 | 99.62 | 7.31 | 3.84 | 2.08 |
| NRC SL-1 | 1830434 | 1822700 | 99.58 | 6 | 4.25 | 1.97 |
| PS 1029 | 1862054 | 1855261 | 99.64 | 7.64 | 3.48 | 1.97 |
| BAUS 96-17 | 2064030 | 2056853 | 99.65 | 7.29 | 3.82 | 2.06 |
| ASB-9 | 1933452 | 1926788 | 99.66 | 6.95 | 3.9 | 2.04 |
| DS 3144 | 1797052 | 1783907 | 99.27 | 6.18 | 4.12 | 2.02 |
| RVS 2012-10 | 2029138 | 2022286 | 99.66 | 6.69 | 4.16 | 2.07 |
| JS 22-14 | 2062914 | 2054317 | 99.58 | 7.29 | 3.95 | 2.1 |
| DLSB 2 | 2170198 | 2159638 | 99.51 | 7.33 | 4.03 | 2.13 |
| AS-15 | 2503922 | 2495426 | 99.66 | 7.79 | 4.36 | 2.37 |
| VLS 101 | 2139056 | 2129104 | 99.53 | 6.72 | 4.44 | 2.23 |
| MAUS 768 | 2297632 | 2290062 | 99.67 | 7.35 | 4.3 | 2.3 |
| MACS 1691 | 1839736 | 1833037 | 99.64 | 5.92 | 4.33 | 2 |
| JS 93-05 | 1996538 | 1988933 | 99.62 | 7.99 | 3.49 | 1.99 |
| MAUS 806 | 2332952 | 2325503 | 99.68 | 7.44 | 4.35 | 2.32 |
| DLSB-1 | 2332278 | 2324460 | 99.66 | 7.54 | 4.24 | 2.29 |
| NRC 109 | 2443126 | 2434751 | 99.66 | 7.36 | 4.47 | 2.36 |
| BAUS 31-17 | 2454596 | 2445927 | 99.65 | 7.17 | 4.6 | 2.37 |
| NRC 128 | 2083610 | 2077194 | 99.69 | 7.51 | 3.76 | 2.05 |
| RVSM 2012-11 | 2220594 | 2170978 | 97.77 | 7.15 | 4.18 | 2.15 |
| DS 3-05 | 2264352 | 2255593 | 99.61 | 7.1 | 4.36 | 2.24 |
| Average | 1887742.23 | 1879552.58 | 99.56 | 6.81 | 3.88 | 1.97 |
