# Supplementary material for: Population structure and genetic diversity characterization of soybean for seed longevity
Source: PLoS One. 2022 Dec 6;17(12):e0278631. doi: 10.1371/journal.pone.0278631 (PMC9725150; doi:10.1371/journal.pone.0278631)
Supplement: S5 Table — (DOCX) [file pone.0278631.s006.docx]

**S5 table. Number and percentage of discovered SNPs in each chromosome out of 26769 chromosome aligned SNPs**

| Chromosome number | Chromosome code | Number of SNPs | Percentage of SNPs per chromosome |
| --- | --- | --- | --- |
| 1 | NC_016088.4 | 985 | 3.68 |
| 2 | NC_016089.4 | 1806 | 6.75 |
| 3 | NC_016090.4 | 1114 | 4.16 |
| 4 | NC_016091.4 | 956 | 3.57 |
| 5 | NC_038241.2 | 1030 | 3.85 |
| 6 | NC_038242.2 | 1933 | 7.22 |
| 7 | NC_038243.2 | 1158 | 4.33 |
| 8 | NC_038244.2 | 1193 | 4.46 |
| 9 | NC_038245.2 | 1148 | 4.29 |
| 10 | NC_038246.2 | 753 | 2.81 |
| 11 | NC_038247.2 | 1027 | 3.84 |
| 12 | NC_038248.2 | 775 | 2.90 |
| 13 | NC_038249.2 | 1207 | 4.51 |
| 14 | NC_038250.2 | 1998 | 7.46 |
| 15 | NC_038251.2 | 2339 | 8.74 |
| 16 | NC_038252.2 | 1199 | 4.48 |
| 17 | NC_038253.2 | 1221 | 4.56 |
| 18 | NC_038254.2 | 2765 | 10.33 |
| 19 | NC_038255.2 | 834 | 3.12 |
| 20 | NC_038256.2 | 1328 | 4.96 |
