# Supplementary material for: Population structure and genetic diversity characterization of soybean for seed longevity
Source: PLoS One. 2022 Dec 6;17(12):e0278631. doi: 10.1371/journal.pone.0278631 (PMC9725150; doi:10.1371/journal.pone.0278631)
Supplement: S6 Table — (DOCX) [file pone.0278631.s007.docx]

**S6 table. Fixation index, expected heterozygosity and number of genotypes in six sub groups of soybean genotypes derived from SNP population structure analysis**

| **Subpopulations** | **Fixation index (Fst)** | **Inferred clusters** | **Expected Heterozygosity (He)** | **No. of genotypes** | **Seed longevity** | **100 Seed weight (g)** | **Plant height (cm)** |
| --- | --- | --- | --- | --- | --- | --- | --- |
| **C1** | 0.353 | 0.189 | 0.294 | 19 | 36.169 | 14.642 | 52.250 |
| **C2** | 0.529 | 0.187 | 0.228 | 18 | 45.816 | 13.788 | 44.702 |
| **C3** | 0.749 | 0.264 | 0.094 | 29 | 39.390 | 14.013 | 38.860 |
| **C4** | 0.627 | 0.138 | 0.184 | 12 | 39.716 | 13.916 | 51.958 |
| **C5** | 0.796 | 0.095 | 0.131 | 7 | 35.347 | 12.917 | 43.435 |
| **C6** | 0.738 | 0.127 | 0.141 | 11 | 49.544 | 11.263 | 67.060 |
